# A monograph of the African and Madagascan species of *Cyperus* sect. *Incurvi* (Cyperaceae)

**DOI:** 10.1007/s12225-022-10058-9

**Published:** 2022-10-26

**Authors:** Megan Griffiths, Hélène Ralimanana, Franck Rakotonasolo, Isabel Larridon

**Affiliations:** 1 Royal Botanic Gardens, Kew, Richmond, Surrey TW9 3AE UK; 2 School of Biological and Chemical Sciences, Queen Mary University of London, Mile End Rd, London, E1 4NS UK; 3 Kew Madagascar Conservation Centre, Lot II J 131 B Ambodivoanjo, 101 Antananarivo, Madagascar; 4 University of Antananarivo, B.P. 906, Antananarivo, Madagascar; 5 Parc Botanique et Zoologique de Tsimbazaza, BP 4096, Rue Kasanga, Antananarivo, Madagascar; 6 Department of Biology, Systematic and Evolutionary Botany Lab, Ghent University, K.L. Ledeganckstraat 35, 9000 Ghent, Belgium

**Keywords:** Conservation, Cyperaceae, *Cyperus*, lectotypification, Madagascar, taxonomy

## Abstract

*Cyperus* sect. *Incurvi* (Cyperaceae) contains 31 species worldwide, with important continental radiations in Australasia, Tropical Africa and Madagascar, and the Neotropics. Here, a monograph of the African and Madagascan species of *Cyperus* sect. *Incurvi* is presented, including descriptions, illustrations, synonymy, notes on habitat and ecology, geographic distribution ranges and conservation assessments. Our results identify eight species of *Cyperus* sect. *Incurvi* endemic to Madagascar, and a further three species native to Tropical Africa. Seven species of *Cyperus* sect. *Incurvi* have been typified herein. Six rare Madagascan endemics are assessed as threatened with extinction.

## Introduction

Sister to the rushes (Juncaceae), the sedges (Cyperaceae) are graminoid plants with complex, compound inflorescences (Semmouri *et al.* 2019). Cyperaceae include over 5600 species across 95 genera, making them the third largest of the monocot families (Larridon *et al.* 2021a). Cyperaceae have an almost cosmopolitan distribution (POWO 2021) with centres of generic diversity across the tropics (Larridon *et al.* 2019). The most species-rich genus of Cyperaceae, *Carex* L. reaches its highest levels of diversity and biomass in temperate regions, e.g. in Canada where an estimated 10% of native vascular plants are sedges (Danylyk & Kricsfalusy 2020). The second largest Cyperaceae genus is *Cyperus* L. with c 964 species (Larridon *et al.* 2021a). Hotspots for *Cyperus* species diversity occur across the tropics and subtropics. The distribution ranges of *Cyperus* species vary from regional endemics with confined distribution ranges, to species with almost cosmopolitan distributions (Kükenthal 1935 – 36; Tucker 2014; POWO 2021).

The high degree of morphological variability within *Cyperus* – in particular, the extreme plasticity demonstrated by the *Cyperus* inflorescence – has meant that evolutionary reconstruction based on morphological data has been notoriously complicated (e.g. Larridon *et al.* 2013). High levels of homoplasy in characters used to classify infrageneric groupings has resulted in numerous contrasting taxonomic opinions, and conflicting classification systems for the genus (Rikli 1895; Britton 1907; Goetghebeur 1998). Traditionally, *Cyperus* species were circumscribed as those possessing spikelets with strongly distichous glumes, and flowers that lack a defined perianth (Larridon *et al.* 2011b). However, since Cyperaceae inflorescences regularly demonstrate reductions and contractions in the number of their floral parts, these characters are observed across many sedge species (Muasya *et al*. 2009a, b). Therefore, an additional character is needed to delimitate the genus, namely the development from a *Cyperus-*type embryo (Semmouri *et al.* 2019; Larridon *et al.* 2021a).

Until recently, the most widely accepted classification of the Cyperaceae family was outlined by Goetghebeur (1998). Besides *Cyperus s.s.* (c. 700 spp.), Goetghebeur recognised 14 segregate genera embedded within the *Cyperus* clade (*Alinula* J.Raynal, *Androtrichum* (Brongn.) Brongn., *Ascolepis* Nees ex Steud., *Ascopholis* C.E.C.Fisch., *Courtoisina* Soják, *Kyllinga* Rottb., *Kyllingiella* R.W.Haines & Lye, *Lipocarpha* R.Br., *Oxycaryum* Nees, *Pycreus* P.Beauv., *Queenslandiella* Domin, *Remirea* Aubl., *Sphaerocyperus* Lye and *Volkiella* Merxm. & Czech; Larridon *et al.* 2011b). The delimitation of these genera was based solely on morphological data. The inclusion of molecular phylogenetic data in these analyses revealed that these genera were nested within *Cyperus s.s.* and consequently the segregate genera were subsumed into *Cyperus s.l.* (Larridon *et al.* 2011b, c, 2013; Bauters *et al.* 2014; Pereira-Silva *et al.* 2020). In the new classification of the Cyperaceae family, based on phylogenomic data (Larridon *et al.* 2021a; Larridon 2022), *Cyperus* is placed in a monogeneric subtribe Cyperinae, within tribe Cypereae of subfamily Cyperoideae.

Within *Cyperus*, there is now a consensus that the *Cyperus* species using C_3_ photosynthesis form a paraphyletic group (subgenus *Anosporum* (Nees) C.B.Clarke or the C_3_ Cyperus Grade), within which a monophyletic clade of C_4_ *Cyperus* species (subgenus *Cyperus* or the C_4_ Cyperus Clade) is nested (Larridon *et al.* 2011b, 2013; Reid *et al.* 2014, 2017; Semmouri *et al*. 2019; Larridon *et al.* 2020, 2021a).

*Cyperus* sect. *Incurvi* Kük. represents a subdivision of *Cyperus* (Larridon *et al.* 2011a, b) characterised by glumes with incurved, mucronate apices that “articulate at their saccate, and persistent bases” (Kükenthal 1935 – 36; Larridon *et al.* 2011a). *Cyperus* sect. *Incurvi* falls into *Cyperus* subgenus *Anosporum* (Larridon *et al.* 2011b, c). Molecular phylogenetic analysis placed the section within Clade 1 of the C_3_ Cyperus Grade, along with sections *Diffusi* and *Haspani* (Larridon *et al.* 2011b). This result was recently confirmed by a phylogenomic study (Larridon *et al.* 2021a). Section *Incurvi* consists of 31 species and has a pantropical distribution, with major continental radiations in the Neotropics, Australasia, Tropical Africa and Madagascar (Kükenthal 1935 – 36; Chermezon 1919, 1919 publ. 1920, 1937; Larridon *et al.* 2011b). Table 1 provides an overview of the species currently placed in sect. *Incurvi* along with their distribution.

**Table 1.** Accepted names and distribution ranges (POWO 2021) for all species of *Cyperus* sect. *Incurvi.* The type species is underlined.

**Species**	**Distribution range**
*Cyperus almensis* D.A.Simpson	Brazil
*Cyperus anisitsii* Kük.	Paraguay
*Cyperus consors* C.B.Clarke	SE & S Brazil
*Cyperus dichromenaeformis* Kunth	SE Brazil
*Cyperus grandisimplex* C.B.Clarke	S Venezuela to Paraguay
*Cyperus altsonii* Kük.	N South America, N Peru
*Cyperus inops* C.B.Clarke	S Brazil
*Cyperus lundellii* O'Neill	Mexico to Guatemala
*Cyperus miliifolius* Poepp. & Kunth	C & S Trop. America
*Cyperus pearcei* C.B.Clarke	Peru to Bolivia
*Cyperus simplex* Kunth	S Mexico to Trop. America
*Cyperus subcastaneus* D.A.Simpson	Brazil
*Cyperus disjunctus C.B.Clarke	E Australia
*Cyperus filipes* Benth.	New South Wales
*Cyperus longistylus* Kük.	Solomon Is.
*Cyperus neoguinensis* Kük.	New Guinea
*Cyperus pedunculosus* F.Muell.	New Guinea to N Australia
*Cyperus semifertilis* S.T.Blake	Queensland
*Cyperus subpapuanus* Kük.	Papua New Guinea
*Cyperus tetraphyllus* R.Br.	E Australia
*Cyperus chamaecephalus* Cherm.	E Madagascar
*Cyperus chinsalensis* Podlech	S Tanzania to Zambia
*Cyperus debilissimus* Baker	C Madagascar
*Cyperus fertilis* Boeckeler	W Trop. Africa to Angola
*Cyperus mapanioides* C.B.Clarke	Trop. Africa
*Cyperus molliglumis* Cherm.	C Madagascar
*Cyperus multinervatus* Bosser	Madagascar
*Cyperus pandanophyllum* C.B.Clarke	E Madagascar
*Cyperus plantaginifolius* Cherm.	Madagascar
*Cyperus rufostriatus* C.B.Clarke	E Madagascar
*Cyperus sciaphilus* Cherm.	E Madagascar

**Fig. 1:**
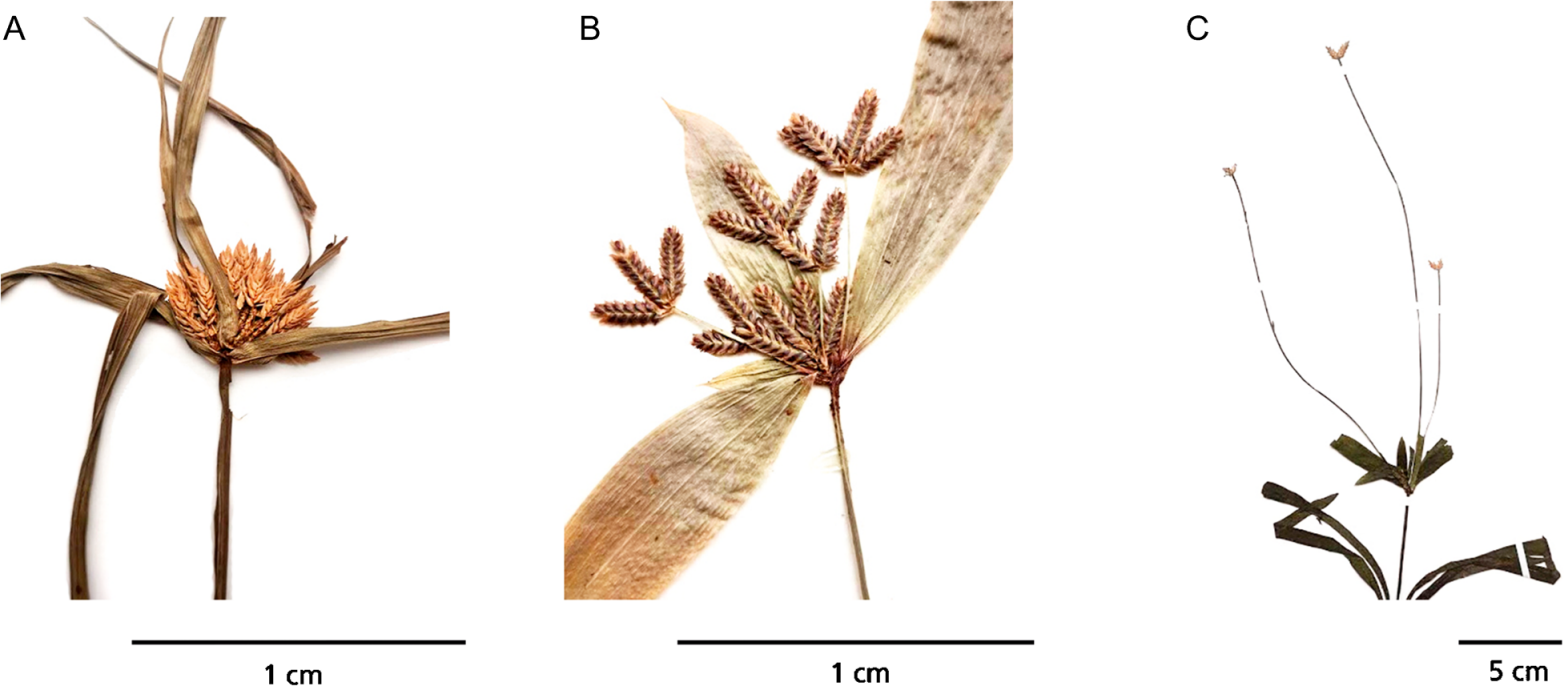
The primary inflorescence types of *Cyperus* sect. *Incurvi*. **A** simple capitate inflorescence, where the internodes of the floral axis have been severely reduced, and spikelets are congested into a capitulum, seen in *C. mapanioides* (*Denys* 1035); **B** anthelate-digitate inflorescence, wherein digitate clusters of spikelets are held atop short rays, seen in *C. sciaphilus* (*Lugd.Bat* 5816); **C** anthelate digitate inflorescence wherein the digitate clusters of spikelets sit atop extensively elongated rays, seen in *C. fertilis* (*Van der Veken* 8940).

Species of sect. *Incurvi* are generally herbaceous perennials with short, woody rhizomes, and trigonous or triquetrous, erect culms (Kükenthal 1935 – 36). Leaves are broadly linear-lanceolate, and are arranged tristichously, with prominent 3-nerved venation running the length of the leaf blade in most species. Several species within the section have leaf blades that fold towards the base to create a narrow, channelled pseudopetiole, above the leaf sheath. Pseudopetioles, and leaf sheaths transition from medium-green to purplish-red at the base in several groups. Involucral bracts are foliate, and subtend the inflorescences. Inflorescences of sect. *Incurvi* are usually reduced to a simple-capitate head but can also be anthelate-digitate (Fig. 1). Spikelets are androgynous, with bisexual flowers that are arranged distichously along the rachilla. Glumes are papery, multi-nerved, with a mucronate apex, and a saccate base. Most species have flowers with three stamens, rarely one, and anthers are usually smooth and sometime setulose. Members of this section typically have trifid styles, and ovate-ellipsoid nutlets, sometimes with a rugulose or papillose surface.

**Fig. 2:**
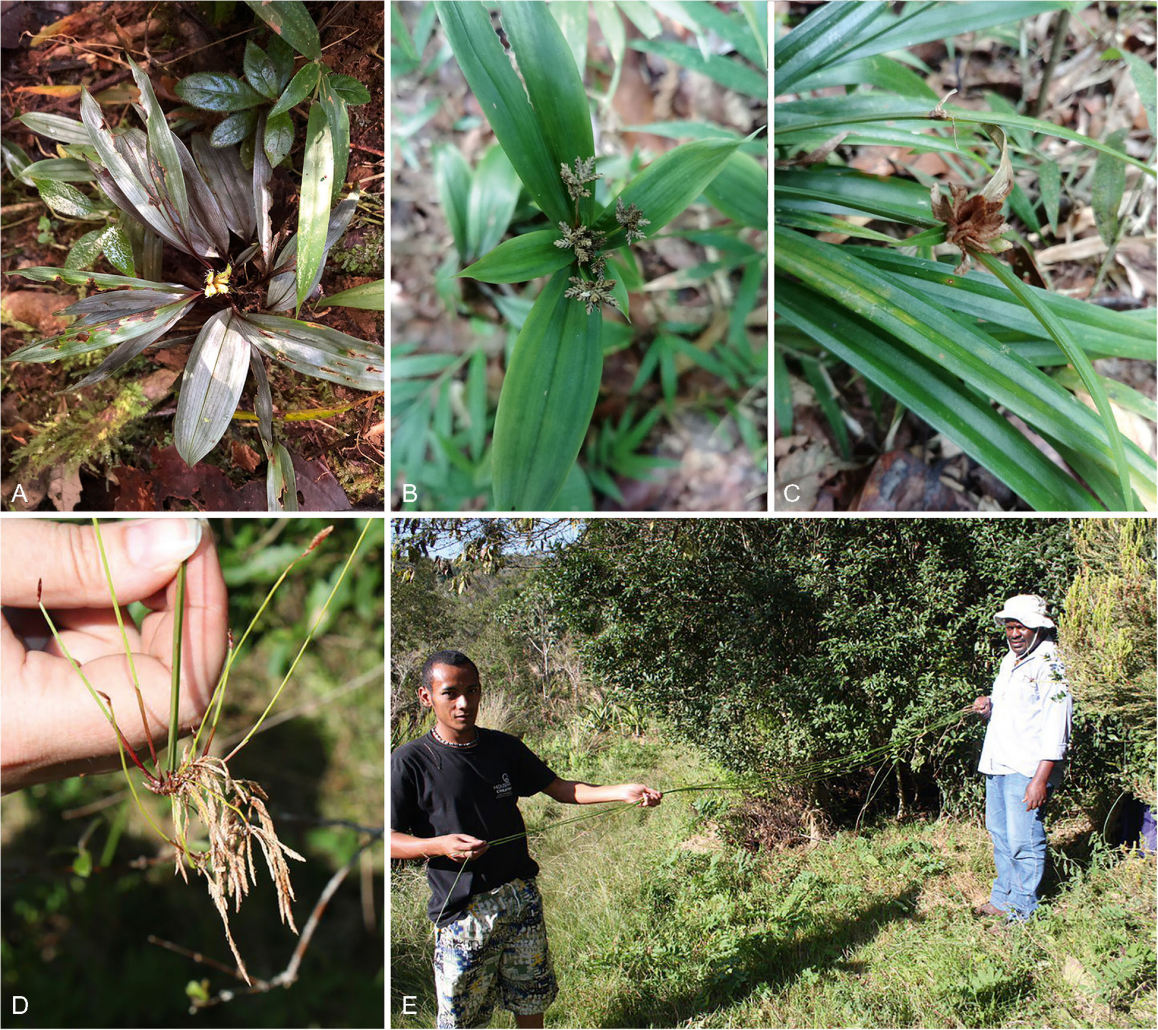
Morphologically remarkable members of *Cyperus* sect. *Incurvi.* **A** forest-dwelling Madagascan endemic species *C. chamaecephalus*, with broad leaves and unusual purple iridescence; **B** broad, leaf-like involucral bracts, and anthelate-digitate inflorescence seen in *C. sciaphilus;* **C** densely capitate inflorescence of *C. molliglumis;* **D** atypical inflorescence structure of *C. debilissimus;* **E** atypical habit of *C. debilissimus,* where the aphyllous culm of the plant shows extreme elongation, up to 300 cm long.

Across the species-rich and morphologically and ecologically diverse genus *Cyperus*, rainforest-dwelling species are rare. Interestingly, the Madagascan radiation of sect. *Incurvi* houses a disproportionate number of species which inhabit the forest floor (Simpson 1992). Their adaptation to this distinct environment has resulted in their atypical morphologies (Fig. 2; Larridon *et al.* 2021b). The Madagascan species of sect. *Incurvi* that inhabit the understorey of tropical forests (*C. chamaecephalus* Cherm., *C. molliglumis* Cherm., *C. pandanophyllum* C.B.Clarke, *C. plantaginifolius* Cherm., *C. rufostriatus* C.B.Clarke) are characterised by their broad leaves, sometimes with purplish iridescence, purple-to red leaf sheaths, channelled pseudopetioles, and long, leaf-like involucral bracts which exceed their simple, capitate inflorescences. These morphological differences were pronounced enough for Chermezon (1937) to place them in the separate *Cyperus* sect. *Pandanophylli* Cherm. The remarkable, aphyllous Madagascan endemic *Cyperus debilissimus* Baker (Fig. 2) was also assigned to its own section, *Cyperus* sect. *Debilissimi* Cherm. by Chermezon (1937). Kükenthal (1935 – 36) placed this species in his sect. *Vaginati* (Boeckeler) Kük., a synonym of the accepted sect. *Alternifolii* (Kunth) C.B.Clarke (Larridon *et al.* 2011a). However, the molecular phylogenetic study of Larridon *et al.* (2011b) placed *C. debilissimus* alongside the rainforest-dwelling Madagascan species of sect. *Incurvi*. *Cyperus sciaphilus* was previously grouped with sect. *Diffusi* Cherm. (Chermezon 1937), before Kükenthal’s review of the genus reassigned it to sect. *Incurvi,* on account of possessing incurved, mucronate glumes (Kükenthal 1935 – 36). Table 2 provides an overview of the sectional placement of the African and Madagascan species of sect. *Incurvi*.

**Table 2.** Sectional placement of the African and Madagascan species of *Cyperus* sect. *Incurvi* in literature*.* Where species were previously assigned to different sections, the names are indicated in bold.

**Species**	**Chermezon (**1937**)**	**Kükenthal (**1935**– 36)**	**Larridon** ***et al.*** **(**2011b**)**
*Cyperus chamaecephalus*	**Sect.** ***Pandanophylli***	Sect. *Incurvi*	Sect. *Incurvi*
*Cyperus chinsalensis*	Not assigned	Sect. *Incurvi*	Sect. *Incurvi*
*Cyperus debilissimus*	**Sect.** ***Debilissimi***	**Sect.** ***Vaginati***	Sect. *Incurvi*
*Cyperus fertilis*	Not assigned	Sect. *Incurvi*	Sect. *Incurvi*
*Cyperus mapanioides*	Not assigned	Sect. *Incurvi*	Sect. *Incurvi*
*Cyperus molliglumis*	**Sect.** ***Pandanophylli***	Sect. *Incurvi*	Sect. *Incurvi*
*Cyperus multinervatus*	Not assigned	Sect. *Incurvi*	Sect. *Incurvi*
*Cyperus pandanophyllum*	**Sect.** ***Pandanophylli***	Sect. *Incurvi*	Sect. *Incurvi*
*Cyperus plantaginifolius*	**Sect.** ***Pandanophylli***	Sect. *Incurvi*	Sect. *Incurvi*
*Cyperus rufostriatus*	**Sect.** ***Pandanophylli***	Sect. *Incurvi*	Sect. *Incurvi*
*Cyperus sciaphilus*	**Sect.** ***Diffusi***	Sect. *Incurvi*	Sect. *Incurvi*

While a more complete image of the infrageneric relationships within *Cyperus* is taking shape, there are still sampling gaps for the phylogenetic trees that have been re-constructed for the genus. One such gap is the absence of the South American and Australasian species of sect. *Incurvi* (which includes the type for the genus, the Australian species *Cyperus disjunctus* C.B.Clarke) from any molecular revision to date. The phylogenetic study conducted by Larridon *et al.* (2011b) focused on the Madagascan species of sect. *Incurvi* and included *C. chamaecephalus, C. molliglumis*, and *C. plantaginifolius* (and the Madagascan endemic *Cyperus betafensis* Cherm.*,* previously placed within sect. *Incurvi*, and which has since been reassigned to sect. *Diffusi*). Molecular data are sparse for the other continental radiations of sect. *Incurvi*, and the phylogenetic placement of these geographically distinct lineages remains to be assessed to understand the correct delimitation of the section and the evolutionary relationships within it.

Since the taxonomic overview by Kükenthal (1935 – 36), in which the section was established, and the *Flore de Madagascar* by Chermezon (1937) which compiled descriptions and a key for the Madagascan species, most species of sect. *Incurvi* have received very little taxonomic scrutiny. Consequently, almost all the Madagascan species of the section are yet to be adequately described (in English), and no assessment of their conservation status has yet been performed. This study presents a monograph for the African and Madagascan species of sect. *Incurvi*, which will serve to contribute towards a growing revision of the Cyperaceae from Africa and Madagascar (Bauters *et al.* 2019; Galán Díaz *et al.* 2019; Larridon *et al.* 2019). A taxonomic treatment, including species descriptions, illustrations, distribution maps and conservation assessments according to IUCN guidelines (IUCN 2012) is provided for the species discussed herein.

## Materials & Methods

### Morphological study

Herbarium material held at the Royal Botanic Gardens, Kew (K) and the Ghent University Herbarium (GENT) was examined first-hand by the author (MG). All specimens examined in person are listed in the taxonomic treatment below, and represent the herbarium material available before access to vouchers was restricted due to COVID-19 restrictions. The treatment was subsequently supplemented with digitised specimen records. Morphological traits used to construct the treatment are included in Table 3. Dried specimens were studied using a Leica S6 E stereo microscope, with a magnification up to 40×. Measurements were taken by hand with a standard ruler, or for smaller characters (such as glume, style and nutlet length), using the graticule of the herbarium microscope. All morphological characters that featured in sect. *Incurvi* literature were examined to build the treatment. Characters within each specimen were numbered and several randomised measurements were then taken for each character across all specimens. Range values represent the upper and lower extremes of those character states within the specimens examined.

**Table 3.** Character matrix for all studied morphological traits.

	***C. chamaecephalus***	***C. chinsalensis***	***C. debilissimus***	***C. fertilis***
**Culm (cm)**	1 – 5 × 0.15, erect	41 – 92 × 0.1 – 0.3, strongly triquetrous, scabrid margins	< 300 × 0.1, flexuose	15 × 0.1 – 0.3, trigonous to flat
**Leaves (cm)**	5 –14 × 0.1 – 0.2, lanceolate to ellipsoid	< 55 × 0.5 – 0.6, linear, plicate, acuminate apex	n/a	10 – 17 × 0.1 – 0.13, lanceolate to ellipsoid
**Leaf Sheath (cm)**	n/a	n/a	n/a	n/a
**Pseudopetiole (cm)**	2 – 12 × 0.1 – 0.4, purple striations at base	n/a	n/a	n/a
**Involucral Bracts (cm)**	5 – 8 × 0.7 – 1.5, foliate, spreading	3.5 – 10 × 0.3 – 0.4, foliate	4 × 0.15, inconspicuous	8 – 13 × 0.6 – 1.8, foliate, spreading
**Inflorescence (cm)**	1 – 1.5 × 1, loosely capitate	simple, loosely capitate, 3 – 8 primary rays up to 1.5 cm long, 2 – 5 spikelets per ray	< 5 × 4, anthelate digitate	simple, anthelate, 6 – 9 flexuose primary rays 20 – 30 cm long, 1 – 3 spikelets per ray
**Spikelets (mm)**	5 – 10 × 1 – 2, acutely oblong, 8 – 24 flowers per rachilla	8 – 10 × 4 – 6, broadly ovoid, 10 – 15 flowers per rachilla	3 – 10 × 1 – 2, lanceolate, < 30 flowers per rachilla	3 – 5 × 2 – 3, lanceolate, 8 – 12 flowers per rachilla
**Glumes (mm)**	1.5 – 3 × 1, ovate, densely imbricate, whitish-green	3.5 – 4 × 1.3 – 1.8, loosely imbricate, elliptic	1.5 – 2 × 1, lanceolate, reddish-brown, narrowly imbricate	2.5 – 3 × 2, lanceolate, narrowly imbricate
**Stamens (mm)**	smooth, linear	filaments 1.9 – 3 long, anthers 1 – 1.6 mm long, tip linear	smooth, yellow to white, setulose	1.8 mm long, 3, anther linear and smooth
**Stigmas (mm)**	2.3 – 2.5, 3-branched	style long and exserted, 3-branched	3 mm long, 3-branched, exceeding the glumes	2.8 – 3.2 mm long, exceeding the glumes
**Nutlet (mm)**	0.8 – 1.2 × 0.7, globose, bluntly trigonous	broadly obovate, trigonous, concave, reddish-brown	0.4 – 0.8 × 0.3, strongly triquetrous, light brown	1.8 – 2 × 1, trigonous, ellipsoid with truncated base
	***C. mapanioides***	***C. molliglumis***	***C. multinervatus***	***C. pandanophyllum***
**Culm (cm)**	15 – 50 × 0.14 – 0.39, trigonous, glabrous	5 – 15 cm × 0.1 – 0.2, trigonous	3 – 6 × 0.2, acutely trigonous	15 – 25 × 0.15 – 0.5, strongly trigonous, winged
**Leaves (cm)**	< 40 × 0.4 – 1.2, linear, scabrid at margins, apex acute	20 – 40 × 1 – 1.5, narrowly lanceolate	< 75 × 1 – 2, linear- lanceolate margins scabrid	< 20 × 0.15 – 0.4, lanceolate-ovate
**Leaf Sheath (cm)**	1.5 – 7 cm long, deep purple at base	n/a	n/a	n/a
**Pseudopetiole (cm)**	n/a	6 – 8 × 0.4, purple towards the base	3 – 5 × 0.2, reddish-purple towards base	5 – 10 × 0.5 – 1, tapering, purple at base
**Involucral Bracts (cm)**	10 – 34 × 0.6 – 1.3, foliate, 4 – 7, spreading	5 – 20 × 1.5 – 2, foliate, 3 variable in length	10 – 60 × 0.6 – 2, leaf-like, 3 – 4	< 20 × 0.15 – 0.2, lanceolate, 3, variable in length
**Inflorescence (cm)**	loosely capitate, 7 – 20 spikelets	1.5 × 2.5, densely capitate cluster of many flowers	1.5 – 2 × 1.5 – 2, simple, condensed capitate	1 – 1.5 × 1.5 – 2, simple capitate
**Spikelets (mm)**	7 – 18 × 2.4 – 4, linear-lanceolate to ovoid, 8 – 16 flowers	5 – 9 × 3, ovate lanceolate, obtuse, 8 – 16 flowers per rachilla	15 – 18 × 1 – 2, erect, strongly linear-lanceolate	6 – 10 × 3 – 5, lanceolate-ovate, 16 – 25 flowers per rachilla
**Glumes (mm)**	2.5 – 3 × 2, lanceolate-ovate, whitish- grey, ciliate at margins	3 × 2, densely imbricate, ovate, strongly mucronate	5 – 6 × 2 – 3, densely imbricate, broadly ovate	3 – 4 × 2, densely imbricate, ovate, shortly mucronate
**Stamens (mm)**	anther 1.3 – 3 mm long	anthers linear, tip smooth	3, anthers linear	3, anther tip smooth
**Stigmas (mm)**	0.6 – 1.3 mm long	1 mm long, deeply-branched	strongly 3-branched	stigma 3-branched, style 1.5 mm long
**Nutlet (mm)**	1.4 – 1.9 × 0.9 – 1.3, smooth, sometimes minutely papillose	1.2 – 1.5 × 0.8 – 1, ellipsoid, minutely papillose	1 × 0.7, widely ellipsoid, reddish-brown	2 × 1, trigonous, obtuse, minutely rugolose
	***C. plantaginifolius***	***C. rufostriatus***	***C. sciaphilus***	
**Culm (cm)**	15 – 45 × 0.14 – 0.2, trigonous	5 – 25 × 0.1 – 0.3, 3 winged	20 – 30 × 0.1, triangular and ridged	
**Leaves (cm)**	10 – 25 × 1.5, lanceolate, scabrous	10 – 40 × 0.5 – 1.5, linear to linear-oblong	7 – 15 × 0.15 – 0.2, ovate to lanceolate,	
**Leaf Sheath (cm)**	n/a		n/a	
**Pseudopetiole (cm)**	8 – 15 × 0.2 – 0.4, purplish-red at base	3 – 8 × 2 – 5, mid-brown to dark reddish-purple	2 – 7 × 0.1 – 0.3	
**Involucral Bracts (cm)**	<18 × 1.5, foliate, 3, variable length	<40 × 1.5, spreading outwards	10 × 2, foliate, spreading outwards	
**Inflorescence (cm)**	0.5 – 1.5 × 1.5 – 2, simple, capitate, spherical clusters of 8 – 25 sessile spikelets	1 – 1.5 × 0.5 – 3, simple capitate, bearing 3 – 16 spikelets	2 – 3 × 1.5 – 4, anthelate-digitate, 4 – 6 rays per inflorescence, 2 – 4 spikelets per ray	
**Spikelets (mm)**	5 – 10 × 3 – 4, oblong-ovate, 16 – 28 flowers per rachilla	6 – 16 × 3 – 4, lanceolate, flattened with acute apices	4 – 10 × 2 – 3, central linear or oblong, 6 – 24 flowers per rachilla	
**Glumes (mm)**	1 – 3 × 1 – 1.2, oval-obtuse, papery texture, briefly mucronate	4 – 6 × 2 – 3, densely imbricate, ovate-subacute mucronate	1.5 – 2 × 1, loosely imbricate, spreading, elongated oval	
**Stamens (mm)**	3, anther linear, tip smooth	3, anthers linear, tip smooth	3, anther tip linear, minutely setulose	
**Stigmas (mm)**	1.3 mm long, 3-brached, strongly exserted	3-branched	0.2 mm long, 3-branched, curled back	
**Nutlet (mm)**	widely ellipsoid, trigonous, truncated at base	1.5 – 2 × 1.5 – 2, surface lightly papillose	0.8 – 1.5 × 0.7 – 1, trigonous and dark brown, ellipsoid scales	

Digitised collections from the Muséum national d'Histoire naturelle, Paris (P), Meise Botanic Garden (BR) and the Geneva Herbarium (G) were studied remotely, alongside imaged specimens from K, and the JSTOR Global Plants database (https://plants.jstor.org/), to develop comprehensive descriptions for each species. Species descriptions were built on those originally written by Kükenthal (1935 – 36) and Chermezon (1937), texts were translated from Latin and French respectively, with help from Stearn’s *Botanical Latin* (1992). All terminology and definitions follow Beentje’s *Glossary of Botanical Terms* (2016). Classification for the genus *Cyperus* follows the phylogenetic framework developed by Larridon *et al.* (2011b, c). Where needed, lectotypes were assigned following Turland *et al.* (2018), and represent intact, representative specimen sheets of the original type collections.

Illustrations were drawn for all species observed first-hand by the author (MG), and for the species for which herbarium material could not accessed in person, descriptions have been provided based on digitised specimens. Specimens which best represented each of the defining characters for a species were selected for illustrations. All illustrations were drawn by hand using a 0.05 mm fine liner.

### Distribution mapping

Georeferenced data for all studied species were downloaded from GBIF (Derived dataset GBIF.org (20 April 2021). All records complete with herbarium images were identified to species level by the first author in an effort to reinforce the reliability of the data, and to mitigate the compromising nature of any previous misidentifications made in this taxonomically challenging group. Any specimens which had been misidentified were excluded from the distribution maps, and conservation assessment calculations. This resulted in a filtered matrix of GBIF occurrence data which was then used to generate distribution maps 10.15468/dd.9bmsnc). Given the high degree of morphological variability and uncertainty within the group, species which had been incorrectly identified were excluded from distribution maps, to avoid skewing the data with any further misidentifications carried out remotely.

For herbarium specimens which had not yet been georeferenced, approximate coordinates were recovered using the *Gazetteer to Malagasy Botanical Collecting Localities* (Schatz *et al.* 2020). Distribution maps for each species were generated using QGIS v.3.14 (QGIS Development Team 2020). The data points were mapped onto the broad vegetation regions of Madagascar, which were retrieved from the *Geospatial Conservation Atlas* (Majka & Platt 2009).

### Conservation assessments

Conservation assessments were produced following the guidelines set out in the IUCN Categories and Criteria v.3.1 (2012). To generate threat categories, the minimum Area of Occupancy (AOO) and estimated Extent of Occurrence (EOO) for each species was calculated using GeoCAT (Bachman *et al.* 2011).

## Results

### Morphological study

Morphological examination supports the acceptance of eight distinct Madagascan species of sect. *Incurvi*, and three species which are native to Tropical Africa. Generally, species-level identification of the section is clear, however, some specimens show intermediate character states, and might represent relicts of speciation in progress or hybrids. Ambiguities are found between *Cyperus chamaecephalus* and some individuals of *C. rufostriatus*; between *C. molliglumis* and the less-well-known species *C. multinervatus*; and between *C. plantaginifolius* and *C. pandanophyllum*. Descriptions of how to distinguish these morphologically similar species are provided in the ‘Additional Notes’ of the Taxonomic Treatment. Equally, infraspecific variation in the remarkable species *C. debilissimus* can make its identification problematic. Although Chermezon (1937) alluded to the existence of a morphotype distinct from the typical species, we do not recognise distinct taxa within *C. debilissimus*. The findings of the morphological study are included in the Taxonomic Treatment below and are summarised in Table 3. As no digitised specimens are available for the under-studied *C. multinervatus,* no illustration was drawn for this species. All herbarium records studied are provided below. Seven lectotypifications were made within this study.

### Distribution mapping

**Fig. 3:**
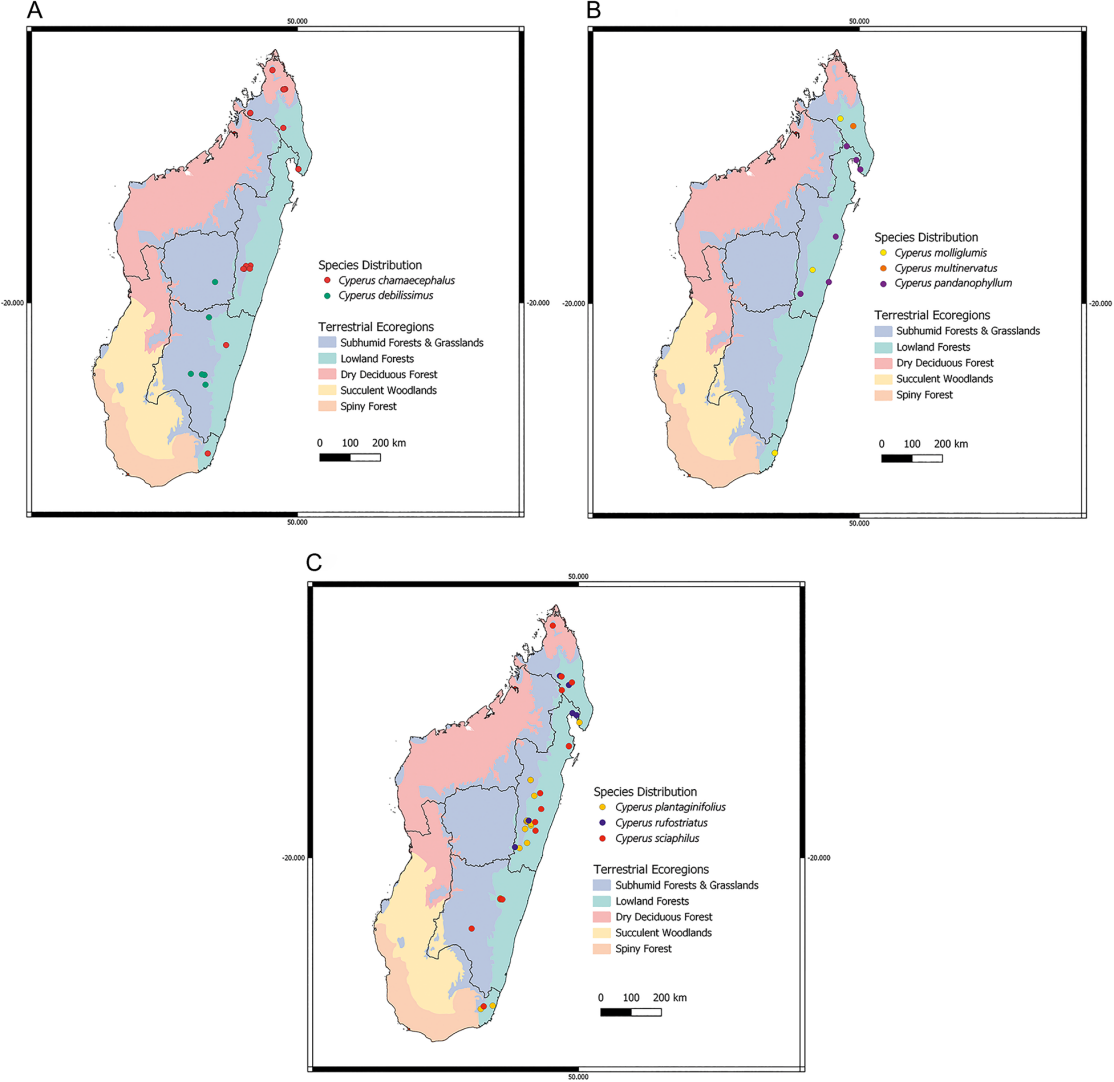
Distribution maps of the eight Madagascan *Cyperus* sect. *Incurvi* species. Points represent georeferenced herbarium vouchers from GENT, K and P, as well as data drawn from GBIF.org (2021). The six provinces of Madagascar are illustrated by black lines. Coloured areas represent Madagascar’s seven broad vegetation zones (Majka & Platt 2009).

While sparse collections of sect. *Incurvi* have been made across the whole length of Madagascar, most vouchers per species were collected from the subhumid forests, and evergreen lowland forests of central and eastern Madagascar (Fig. 3). Distribution mapping indicates a preliminary degree of habitat specialisation in certain Madagascan species. While *C. debilissimus* occurs exclusively in the subhumid forests and grasslands of south-central Madagascar, *C. molliglumis*, *C. pandanophyllum*, *C. plantaginifolius* and *C. rufostriatus* are restricted to lowland forests of eastern Madagascar. However, *C. chamaecephalus* and *C. sciaphilus* show a more generalist habitat preference and are found across multiple ecoregions on the island. All findings must be considered in the light of evident sampling bias in the data used to generate our maps, with many collections clustering in easily accessible areas, near roads and within the limits of national parks. We also acknowledge that scarcity of data doesn’t necessarily equate to rarity, and could simply be a relic of under sampling. However, despite these deficiencies, the pronounced lack of distribution data for *Incurvi* species limits the options available for precise occurrence mapping in this group. We therefore conclude that this method is appropriate for building a preliminary understanding of the occurrence patterns of sect. *Incurvi* across Africa and Madagascar, but note that future research on this group should include field sampling to generate the most accurate abundance and distribution maps possible.

**Fig. 4:**
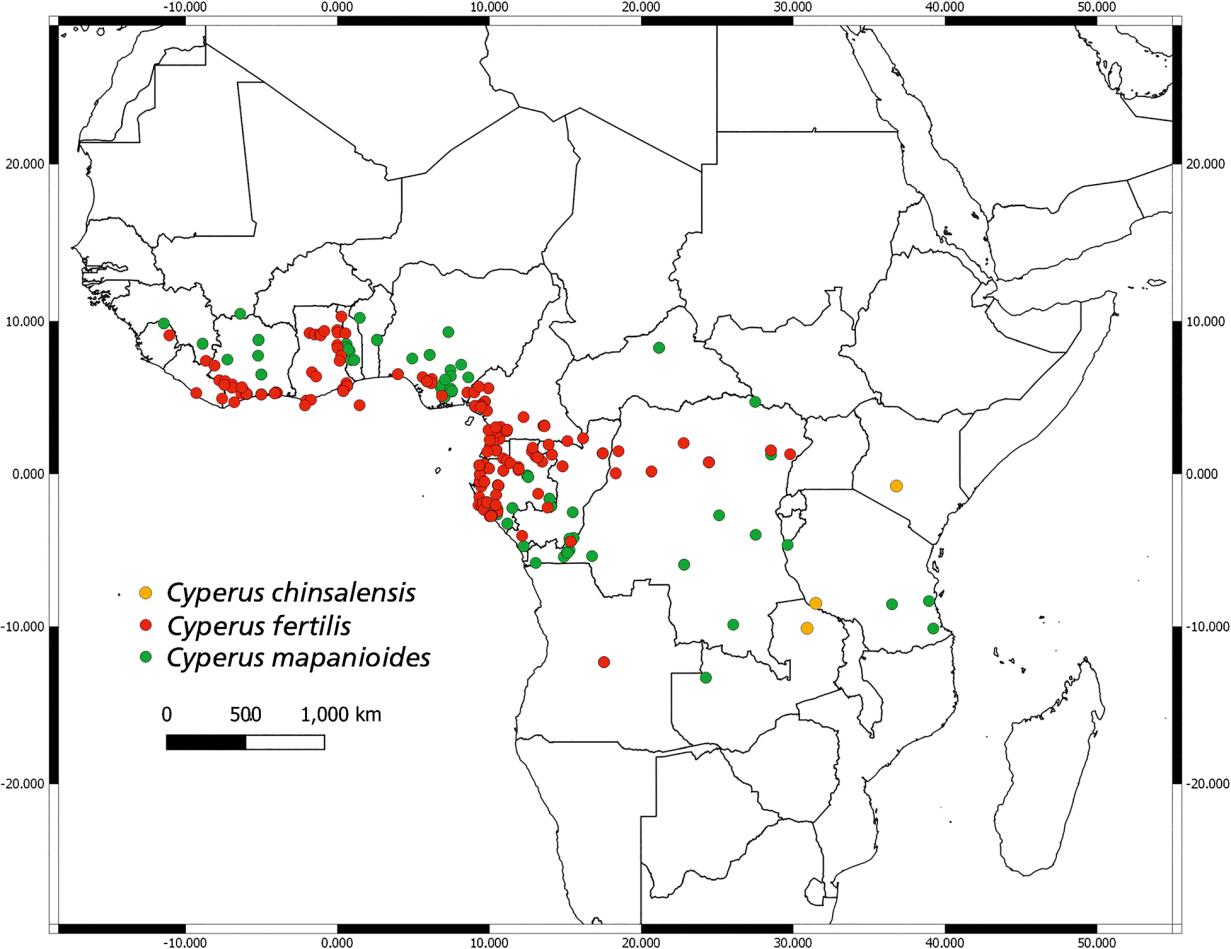
Distribution ranges for the three Tropical African species of *Cyperus* sect. *Incurvi.*

Tropical African species of sect. *Incurvi* have much broader distribution ranges than the Madagascan taxa (Fig. 4), extending across the breadth of sub-Saharan Africa. *Cyperus fertilis* and *C. mapanioides* are the most extensively collected species of the section, and both species are native to West Tropical Africa. The little-known African species, *C. chinsalensis*, has a scattered and patchy distribution across East Africa, ranging from Tanzania to Kenya.

### Conservation assessments

Of the eight Madagascan species of sect. *Incurvi*, six are assessed as threatened with extinction. Four of these threatened species are evaluated as Endangered (EN), with three assessed as EN B2ab(i,ii), due to their limited geographic ranges (AOO <500 km^2^), limited number of locations, and declining extent of habitat (*C. debilissimus*, *C. pandanophyllum*, *C. rufostriatus). Cyperus molliglumis*, is assessed as EN B1ab(i,ii,iii,iv) + EN B2ab(i,ii,iii,iv) as a result of its severely restricted EOO (EOO = 2346 km^2^ <5000 km^2^) and only being known from three georeferenced herbarium records. Two of the threatened species (*C. chamaecephalus* and *C. plantaginifolius*) are assessed as Vulnerable VU B2ab(i,ii), given their limited, but not severely restricted ranges (AOO <2000 km^2^, but EOO >20,000 km^2^, above the threshold for EN). The Tropical African species *C. chinsalensis* was assessed as VU D2 given its severely restricted AOO (12 km^2^) and the limited estimated number of locations (3). Three species (the Tropical African species *C. fertilis* and *C. mapanioides*, and the Madagascan *C. sciaphilus*) were assessed as Least Concern (LC) due to their wide geographic ranges, and non-specific threats at this time. The final Madagascan species of sect. *Incurvi*, *C. multinervatus*, known only from a single georeferenced herbarium record, was not assessed, and falls into the Data Deficient (DD) category, due to a lack of information about its distribution, habitat preference, life history and potential threats.

As with the distribution maps, the sampling deficit present for Cyperaceae species will mean that the AOO and EOO calculated here are unlikely to be entirely accurate, which may inflate IUCN rankings. Moreover, the standard grid cell size recommended by the IUCN has been shown to overestimate the threat assessment when based on specimen data alone (Callmander *et al.* 2007). However, given under sampling, options for precise occurrence mapping are severely limited and we conclude that this method is appropriate for developing a preliminary understanding of the conservation status of sect. *Incurvi* species.

## Discussion

### Taxonomy and morphology of the Afro-Madagascar species of sect. *Incurvi*

Several recent studies in Cyperaceae have re-assigned the taxonomic limits of widely distributed, polyphyletic groups, to contain several smaller, monophyletic taxonomic entities, with narrower geographic ranges (e.g. Larridon *et al.* 2018, 2021c; Barrett *et al.* 2019, 2020, 2021a, b). These larger groupings were based on shared morphology. However, high levels of homoplasy within certain groups of Cyperaceae compromise the reliability of these groups without the inclusion of molecular phylogenetic data. *Cyperus* sect. *Incurvi* is one such pantropical grouping, circumscribed by a single character state (the presence or absence of an incurved apex on the floral glumes; Kükenthal 1935 – 36). To assess whether this shared character is indicative of shared ancestry (synapomorphic) or is simply a relic of convergent evolution across separate continents (homoplastic), an in-depth molecular phylogenetic or phylogenomic study including species of sect. *Incurvi* from the Neotropics, Australasia, Tropical Africa and Madagascar, in addition to species from the other sections placed in Clade 1 of the C_3_ Cyperus Grade (*Diffusi* and *Haspani*) is required. Larridon *et al.* (2011b) stated that *Cyperus* sect. *Incurvi sensu* Kükenthal (1935 – 36), is likely to be heterogenous. Preliminary data show that a Brazilian species of sect. *Incurvi* is not directly related to the Madagascan species (Pereira-Silva *et al.* unpubl. data). This finding provides tentative support that sect. *Incurvi* may indeed be polyphyletic.

Chermezon (1937), in which the first and only key for the Madagascan species of sect. *Incurvi* was published, advocated for the classification of the Madagascan lowland forest species as their own distinct taxonomic entity, sect. *Pandanophylli*. He designated this grouping on the basis of shared morphology and phenotypic distinctiveness from the other members of sect. *Incurvi*. Currently, three lowland forest species from Madagascar have been sequenced (*C. chamaecephalus*, *C. molliglumis* and *C. plantaginifolius*) which together form a clade sister to *C. debilissimus*, a species restricted to the subhumid forests and grasslands of south-central Madagascar (Larridon *et al.* 2011b). The latter species was previously placed in what is now sect. *Alternifolii* (Kunth) C.B.Clarke (previously sect. *Vaginati* (Kukenthal 1935 – 36) on the basis of its vegetative morphology, which is distinct from the other species of sect. *Incurvi* that have adapted to grow in forest understorey. If future studies resolve sect. *Incurvi* as polyphyletic, these results could provide preliminary support towards a monophyletic sect. *Pandanophylli* expanded to include *C. debilissimus*.

It is now widely agreed that many of the morphological traits previously used to classify genera of Cyperaceae, and those of many other vascular plant families, are not phylogenetically informative (Larridon *et al.* 2013). This has been demonstrated in several studies where, in the light of molecular data, groupings initially based on morphological similarity result in paraphyletic scattering of taxa across multiple groups. This is exemplified in the re-circumscription of paraphyletic or polyphyletic Cyperaceae taxa (e.g. in *Cyperus*: Larridon *et al.* 2011b, 2013; Bauters *et al.* 2014; and in *Carex*: Global Carex Group 2015), as well as the reclassification of giant genera from other vascular plant families (Miller & Seigler 2012 in *Acacia* Mill.; Berry *et al.* 2005 in *Croton* L.), or even at family level (e.g. the circumscription of Scrophulariaceae; Oxelman *et al.* 2005). Conversely, taxa previously considered distinct on morphological grounds have been found to be closely related after molecular analysis and examination of more phylogenetically informative morphological traits, such as embryo morphology. The close evolutionary relationship discovered between *Cyperus polystachyos* Rottb. (type species for the segregate genus *Pycreus* P.Beauv.), and *Cyperus laevigatus* L. (type species for the segregate genus *Juncellus* C.B.Clarke) exemplifies how contrasting morphology may not reflect independent evolutionary history within Cyperaceae, but rather rapid adaptation and diversification (Larridon *et al.* 2013, 2020). Similar examples can be seen outside Cyperaceae, such as the African Eriocaulaceae genus, *Mesanthemum* Körn (Liang *et al.* 2019), and in the re-circumscription of the Dioscoreales to include the families Burmanniaceae and Thismiaceae in light of morphological synapomorphies which had not previously been considered relevant when constructing the taxonomy of the higher systematic levels of this order (Caddick *et al.* 2002a, b). These types of studies emphasise the importance of integrating molecular and morphological information to identify which traits are phylogenetically informative. In line with these studies, the traits used to define sect. *Incurvi* and its two most closely related sections *Diffusi* and *Haspani* may not reflect independent evolutionary history, and extensive research utilising ‘a total evidence approach’ will be required to reveal whether the presence or absence of incurved glumes is sufficient to successfully allocate *Cyperus* species to sect. *Incurvi*.

### Distribution and conservation of sect. *Incurvi* in Madagascar

Of the eight Madagascan endemic species of sect. *Incurvi*, six are assessed as threatened (75% of the endemic species of sect. *Incurvi*). Their vulnerability to extinction relates to their narrow distribution ranges, and loss of their preferred habitat under anthropogenic pressures. Similar threat statistics are seen for other Cyperaceae groups of similar size, such as *Costularia*, which includes 11 Madagascan species, eight of which (73%) of which were assessed as threatened (Larridon *et al.* 2019). Like the Madagascan species of sect. *Incurvi*, species of *Costularia* have narrow distribution ranges making them less resilient to stochastic environmental changes and habitat degradation due to human activities. Madagascan Cyperaceae with wider geographic distributions such as some *Scleria* species are buffered from these pressures, resulting in less concerning threat statistics. *Scleria* includes 25 species in Madagascar, only three (12%) of which were assessed as threatened (Galán Díaz *et al.* 2019).

Several common primary threats affect species of sect. *Incurvi* across Madagascar, namely, agricultural expansion by slash-and-burn (*tavy)* agriculture (Kari & Korhonen-Kurki 2013; Desbureaux & Damania 2018), habitat degradation through forest exploitation (e.g. logging and charcoal production), and competition with introduced invasive species (Brown & Gurevitch 2004). Secondary threats impact particular species, including mining (Phillipson *et al.* 2010), conversion of wetlands to rice paddies (Bamford *et al.* 2017), urban development, and drought intensification as a result of climate change (Desbureaux & Damania 2018). Based on the observed decline in coverage of the eastern lowland forests, from satellite imagery and from vegetation maps (Du Puy & Moat 1996), we infer many of the species of sect. *Incurvi* native to the eastern escarpment of Madagascar are at risk of further range restrictions, in terms of both AOO and EOO, in the near future.

Central to developing a strong foundation on which to inform targeted conservation strategies is the need to conduct on-going studies into the population dynamics, ecology, and life history traits of species of sect. *Incurvi*. At present, no quantitative data is available on the health of the population of any of the species of sect. *Incurvi*. Without this information, species-specific conservation action plans cannot be elaborated, and our suggestions are limited to broad-scale, generic action plans.

Central to conserving the Madagascan species of sect. *Incurvi* is the concept of genetic resilience, because their vulnerability to extinction directly relates to their fragmented and narrow distribution ranges. Madagascan species of sect. *Incurvi* are not currently represented in any *ex situ* conservation programmes such as seed banks or living collections (MSB 2021; BGCI 2021). In order to effectively use *ex situ* collections for conservation, the genetic material stored must reflect the genetic diversity of that species (Volis 2016). Consequently, meticulous field work harvesting and storing the germplasm of the species of sect. *Incurvi* is required to ensure there is sufficient genetic material in seed banks for use in any potential *in situ* re-introductions or in ‘forest gene banks’, wherein an existing population is used as a sink into which genetic material from multiple populations is translocated and maintained (Uma Shaanker & Ganeshaiah 1997; Volis 2016). These sink populations act as reservoirs of genetic material, while simultaneously facilitating genetic exchange under protected, natural conditions. The offspring of these sink populations should theoretically harbour greater genetic diversity, which can then be used to bolster existing populations with greater genetic resilience. From these species-specific gene banks, translocation of each species back into its preferred habitat could serve to expand the narrow geographic ranges of the Madagascan species of sect. *Incurvi*, conferring some degree of ecological resilience into these otherwise fragile populations.

Habitat protection is key to plant conservation because it maintains the ecological conditions necessary for the long-term survival of a species. Madagascar now has over 100 protected areas, many of which are managed by the parastatal *Association National pour la Gestion des Aires Protegee* (ANGAP). Despite questions being raised over the effectiveness of the protected areas in Madagascar, Desbureaux & Damania (2018) found that these reserves are successful at limiting the upsurges of deforestation in the parks, even if they are less effective against more inconspicuous activities such as logging and mining. Improving the resources available to protected areas and continuing to train staff in effective protected area management will be essential to safeguarding the diversity that remains within the surviving refuges of Madagascan forests.

Conservation planning in Madagascar needs careful consideration given the complex interplay that exists between biodiversity protection, ecosystem service maintenance, sustainable development, and economic growth (Rakotomanana *et al.* 2013). As poverty is the primary driver of deforestation in Madagascar, we predict that many of the threats to species of sect. *Incurvi* are likely to intensify in the wake of the COVID-19 crisis. A significant proportion of the country’s annual revenue is generated by (eco-) tourism, and as such it will take several years for the country to compensate for the financial losses suffered by halting overseas travel for duration of the lock-down period. This may force an even greater number of people to exploit natural resources, putting an ever-greater pressure on the remaining stretches of intact Madagascan forest. Moreover, many of the protected areas in Madagascar rely heavily on financial aid from foreign countries, and with these countries facing economic uncertainly of their own, it is unclear what this will mean for conservation efforts and the fight against deforestation in Madagascar. A large proportion of the herbarium records of sect. *Incurvi* were collected within the protected area network, but with many parks and reserves unable to pay their conservation agents, the efficacy of the protection in these areas is likely to decline, putting the Madagascan species of sect. *Incurvi* at greater risk.

As a caveat, we should acknowledge that sampling biases, paired with the destruction of many primary habitats in Madagascar since their original collections were made, and the sampling deficit present for Cyperaceae species in general, will mean that the AOO and EOO calculated here are unlikely to be entirely accurate reflections of the true geographic ranges of these species. Moreover, the standard grid cell size recommended by the IUCN has been shown to overestimate the threat assessment when based on specimen data alone (Callmander *et al.* 2007). However, despite these deficiencies, the pronounced lack of distribution data for *Incurvi* species limits the options available for precise occurrence mapping in this group. We therefore conclude that this method is appropriate for building a preliminary understanding of the occurrence patterns of sect. *Incurvi* across Africa and Madagascar, but note that future research on this group should include field sampling to generate the most accurate distribution mapping possible.

### Distribution and conservation of sect. *Incurvi* in Tropical Africa

Unlike the Madagascan members of sect. *Incurvi*, the Tropical African species exhibit broad distribution ranges across Sub-Saharan Africa. Only one of the three African species of sect. *Incurvi* is assessed as threatened, (*C. chinsalensis*) a threat statistic which correlates to its narrow distribution compared to the two other species from Tropical Africa (*C. fertilis* and *C. mapanioides)*. As the two species from West Tropical Africa (*C. fertilis* and *C. mapanioides*) are both assessed as least concern, and both are already represented in *ex situ* collections (BGCI 2021), no species-specific conservation plans need to be drawn up or implemented for them at this time. In contrast, *C. chinsalensis* is only known from three locations, and is yet to be included in any *ex situ* conservation programmes (BGCI 2021). Therefore, intervention may be necessary to augment the ecological resilience of this species, and it should be prioritised for *ex situ* seed conservation. One of the herbarium specimens of *C. chinsalensis* was collected from the Tana River basin, an area of huge socioeconomic importance in Kenya in terms of water provision, hydropower, and agricultural productivity. The river’s health is threatened by anthropogenic pressures such as poor land use management, conversion of savannah and wetlands into agricultural land, soil erosion and over-grazing (Botzen *et al.* 2015). Similar to the situation in Madagascar, progress and socioeconomic benefits in some areas along the Tana River come at a trade off against conservation efforts in others, meaning planning needs to be carefully implemented in a way that protects nature without detracting from human wellbeing. We propose that by designating protected areas along the Tana River delta, in which forest gene banks can be established for the long-term safeguarding of genetic material, a source population of genetic diversity will be created from which future translocations into suitable habitats within the native distribution range can be made.

## Taxonomic Treatment

### Key to the African and Madagascan species of *Cyperus* sect. *Incurvi*

- 1. Inflorescence anthelate-digitate......................................................................................................................................2
- Inflorescence capitate......................................................................................................................................................4
- 2. Spikelets on long, flaccid stem-like peduncles <20 cm long........................................................................**C. fertilis**
- Spikelets sessile or on short peduncles <5 cm long.....................................................................................................3
- 3. Culms extensively elongated (>75 – 300 cm long), flexuose; leaves reduced; involucral bracts inconspicuous; encircling sheaths pronounced, brown..................................................................................................**C. debilissimus**
- Culms rigid, erect (<75 cm long); leaves and involucral bracts conspicuous; sheaths papery and inconspicuous................................................................................................................................................**C. sciaphilus**
- 4. Culms <6 cm long.............................................................................................................................................................5
- Culms >6 cm long.............................................................................................................................................................6
- 5. Leaves long (>15 – 75 cm) margins scabrid, spikelets linear lanceolate, erect................................**C. multinervatus**
- Leaves short (<15 cm) margins smooth; spikelets oblong, partially obscured by subtending bracts...................................................................................................................................................................................**C. chamaecephalus**
- 6. Leaf blades linear.............................................................................................................................................................7
- Leaf blades not linear.......................................................................................................................................................8
- 7. Culm margins scabrid; involucral bracts <10 cm long.........................................................................**C. chinsalensis**
- Culm 3-winged, margins smooth; involucral bracts (>10 – <40 cm) long............................................**C. rufostriatus**
- 8. Leaves exceeding culm by > 15 cm; glumes imbricate, spreading when mature................................**C. mollilgumis**
- Leaves not significantly longer than the culm (< 15 cm longer); glumes densely imbricate, not spreading..........................9
- 9. Leaves rough on both sides, no prominent 3-nerved venation................................................**C. pandanophyllum**
- Leaves smooth on both sides, prominent 3-nerved venation running the length of the leaf blade..................................................................................................................................................................................................................**C. plantaginifolius**

**Cyperus chamaecephalus** *Cherm.* (Chermezon 1925a: 20). Type: Madagascar, Moramanga, Andevorante, 18 Oct. 1912, *Afzelius* s.n. (holotype S-G! [S-G 6066]; isotype S05-11182).

**Fig. 5:**
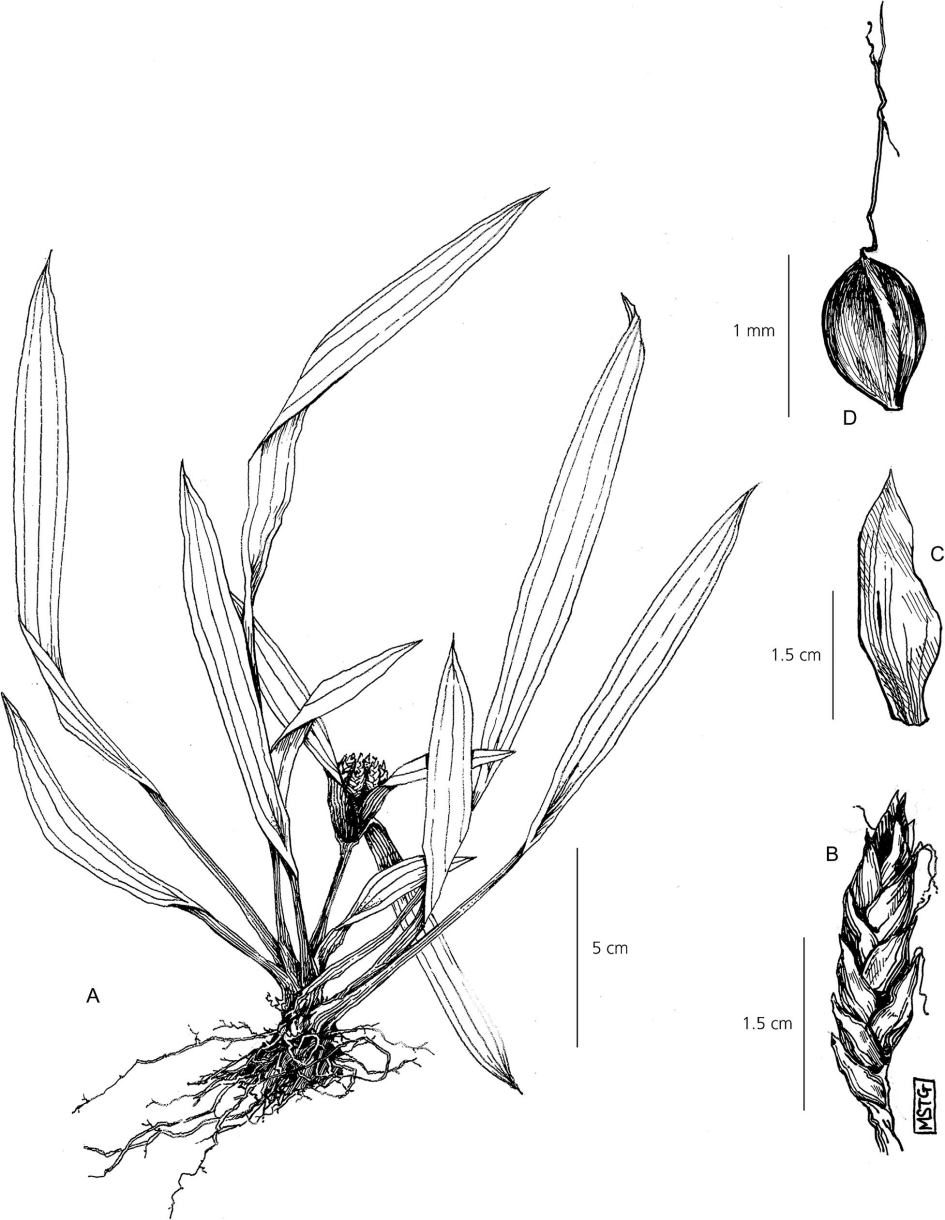
*Cyperus chamaecephalus*. **A** habit; **B** spikelet; **C** glume; **D** nutlet. **A** from *Gautier* 3536; **B, C, D** from *Beentje* 4774. drawn by m. griffiths.

Loosely tufted perennial herb. *Rhizomes* short. *Culms* erect, fairly rigid, 1 – 5 cm × 1.5 mm, strongly triquetrous and glabrous. *Leaves* lanceolate to ellipsoid, far exceeding the culm, c. 5 – 14 × c. 1 – 2 cm, with 3 prominent veins running the length of the leaf, sometimes deep purple with iridescence dependent on environmental conditions. Leaf veins converging and margins folding proximally to create a sheathed pseudopetiole above the leaf sheath, 2 – 12 cm × 1 – 4 mm, transitioning to purple at the base. *Involucral bracts* leaf-like, 2 – 4, c. 5 – 8 × 0.7 – 1.5 cm wide, spreading. *Inflorescence* loosely capitate, 1.5 cm in diam., comprising clusters of 4 – 6 sessile spikelets. *Spikelets* acutely oblong, 0.5 – 1 cm × 1 – 2 mm, bearing 8 – 24 flowers, either partially or completely obscured by the subtending bracts. *Glumes* 1.5 – 3 × 1 mm, densely imbricate and strongly distichous, ovate and membranous, slightly mucronate, dull whitish-green. *Stamens* 3, anthers linear and smooth. *Style* deeply 3-branched 2.3 – 2.5 mm long. *Nutlet* globose 0.8 – 1.2 × 0.7 mm, widely ellipsoid, bluntly triquetrous, finely papillose surface, deep reddish-brown at maturity. Fig. 5.

**distribution**. *Cyperus chamaecephalus* is endemic to the lowland montane rainforests of Madagascar. Individuals of this species have been recorded from the Montagne D'Ambre, and the humid massifs of the Daraina region in the northerly province of Antsiranana, as well as Toamasina, Fianarantsoa and Toliara.

**specimens examined.** **madagascar**: Toamasina, Alaotra-Mangoro, Andevoante [18°56'20"S, 48°13'40"E], 18 Oct. 1912, *Afzelius* s.n. (P); Montagne d'Ambre, partie centrale, 1160 m, [49°09'54"E, 12°36'45"S], 6 Nov. 2007, *Gautier* 5141 (G); Ambahatra, Ridge between the two arms of Ambahatra, [48°25'44"E, 13°59'53"S], 13 March 1999, *Gautier* 3556 (G); Antsiranana, SAVA, Loky Manambato, Daraina, 862 m, [13°13'07"S, 49°35'50"E], 13 Dec. 2005, *Nusbaumer & Ranirison* 1780 (G); Diego-Suarez, Antsiranana, Daraina, Antsahabe forest, 1070 m, [49°32.88'E, 13°12.45'S], 29 Nov. 2004, *Nusbaumer & Ranirison* 1294 (G); Toamasina, Masoala Peninsula, 3 km NE of Antalavia, 200 – 380 m, [50°02'E, 15°47'S], 13 – 16 Nov. 1989, *Schatz* 2789 (MO).

**habitat**. *Cyperus chamaecephalus* occurs in the undergrowth of lowland and montane rainforests, at altitudes between 300 – 1200 m. There are several reports of specimens growing in deep shade, among the humid leaf litter of the forest floor.

**conservation status**. This species is endemic to Madagascar and is only known from eight locations on the island. The estimated AOO (90 km^2^) of *Cyperus chamaecephalus* is below the threshold for VU B2. Despite six of these locations occurring within the protected area network, both the AOO and EOO of the species is forecast to decline as a result of forest exploitation for charcoal production, and agricultural expansion. We therefore assess *C. chamaecephalus* as Vulnerable VU B2ab(i,ii).

**notes**. The atypical inflorescence type in this species has important implications for the reproductive ecology of the plant. Considering the culm of *Cyperus chamaecephalus* is only a few centimetres long, Simpson (1992) emphasised the improbability of wind acting as a dispersal mechanism for pollen. This opens up the likelihood of either autogamy or insect pollination in this species. Further research is needed to confirm this hypothesis.

**Cyperus chinsalensis** *Podlech* (1961: 107). Type: Zambia, 42 km S of Chinsali, *Robinson* 3207 (holotype M! [M-0106894]; isotype K! [K000362643]).

**Fig. 6:**
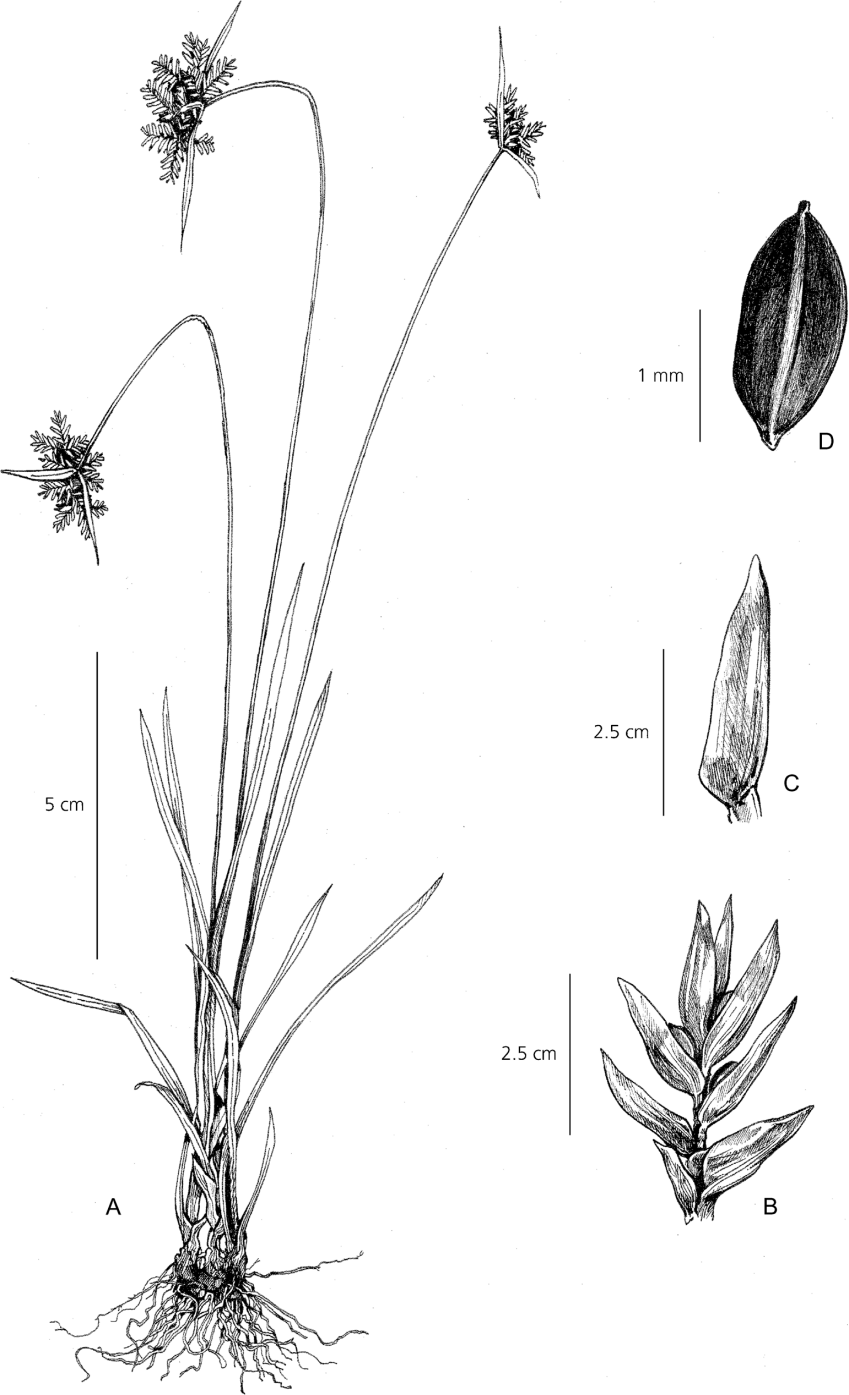
*Cyperus chinsalensis*. **A** habit; **B** spikelet; **C** glume; **D** nutlet. All from *Robinson* 3207. drawn by m. griffiths.

Herbaceous perennial. *Rhizomes* woody and creeping. *Culms* 41 – 92 cm × 1.8 – 3 mm strongly triquetrous with scabrid margins. *Leaves* far exceeded by the culms < 55 cm × 5 – 6 mm, strongly linear, finely plicate, with an extended acuminate apex. *Leaf sheaths* 2 – 7 cm long, papery, pale brown to greenish-brown. *Involucral bracts* 2 – 4, foliate, 3.5 – 10 cm × 3 – 4 mm, spreading outwards. *Inflorescence* simple, loosely capitate, 3 – 8 clusters per inflorescence held atop short rays of unequal length up to 1.5 cm long. *Spikelets* gathered in loose clusters, 2 – 5 spikelets per cluster, broadly ovoid, 8 – 10 × 4 – 6 mm, bearing 10 – 15 loosely imbricate flowers, rachilla finely winged. *Glumes* elliptic, glabrous 3.5 – 4 × 1.3 – 1.8 mm, loosely arranged, spreading when mature, white to straw-coloured, clear venation running the length of the glume. *Stamens* 3, filaments 1.9 – 3 mm long, anthers 1 – 1.6 mm long, tip linear. *Style* long and exserted, 3-branched. *Nutlet* broadly obovate, trigonous, smooth, deep reddish-brown, concave. Fig. 6.

**distribution**. *Cyperus chinsalensis* is native to the rocky granite mountains and Miombo woodlands of Tanzania and Zambia, at elevations between 1300 and 2100 m. Collections for this species have also been made from Zimbabwe, and it is thought to have naturalised as far north as Kenya (Fig. 4).

**specimens examined.** **tanzania**: Rukwa, Ufipa Distr., Mid to upper slopes of Mbaa Mountain, 600 – 6800 ft above Tatanda, 16 Nov. 1986, *Goldblatt et al.* 8131 (MO). **zambia**: 26 miles S of Arinschi, 1341 m, 14 Jan. 1959, *Robinson* 3207 (K); Chishimba Falls, 32 km NW of Kasama, 1380 m, [10°06'19"S, 30°54'54"E], 27 Nov. 1993, *Nkhoma* 45 (MO); 17 Jan. 1964, *Richards* 18810A (BR).

**habitat**. This species has been recorded living on sandy, well drained soils, disturbed areas and rocky granite slopes.

**conservation status**. Given the significantly restricted AOO for this species (12 km^2^) and the limited number of locations it has been found in (3), this species meets the threshold for VU D2. Records indicate that *Cyperus chinsalensis* grows in Miombo woodlands, which are characterised by a dominance of *Brachystegia* species. The loss of primary forest cover in Eastern Africa, driven by shifting cultivation methods, mining and uncontrolled bush fires, is driving the loss of these ecologically important *Brachystegia* populations. Reducing the numbers of these species will alter the community composition of the Miombo woodlands, driving ecological degradation, and putting greater pressure on *Cyperus chinsalensis*. For these reasons we assess *C. chinsalensis* as VU D2.

**Cyperus debilissimus** *Baker* (1887: 532). Type: Madagascar, *R. Baron* 3374 (lectotype K! [K000362684], selected here).

*Cyperus calochrous* Cherm. (Chermezon 1919 publ. 1920: 342). ≡ *Cyperus debilissimus* var. *calochrous* (Cherm.) Cherm. (Chermezon 1927 publ. 1928: 606). Type: Madagascar, Massif de l’Andringitra, 1600 m, Sept. 1911, *H. Perrier de la Bâthie* 2521 (lectotype **designated here**: P! [P00450567]; isolectotype: P00450566).

*Cyperus debilissimus* var. *triqueter* Cherm. (Chermezon 1921: 553) Type: Madagascar, Massif du Manongarivo, April 1921, *H. Perrier de la Bâthie* 13739 (holotype: P00450568, isotype: P00450569).

**Fig. 7:**
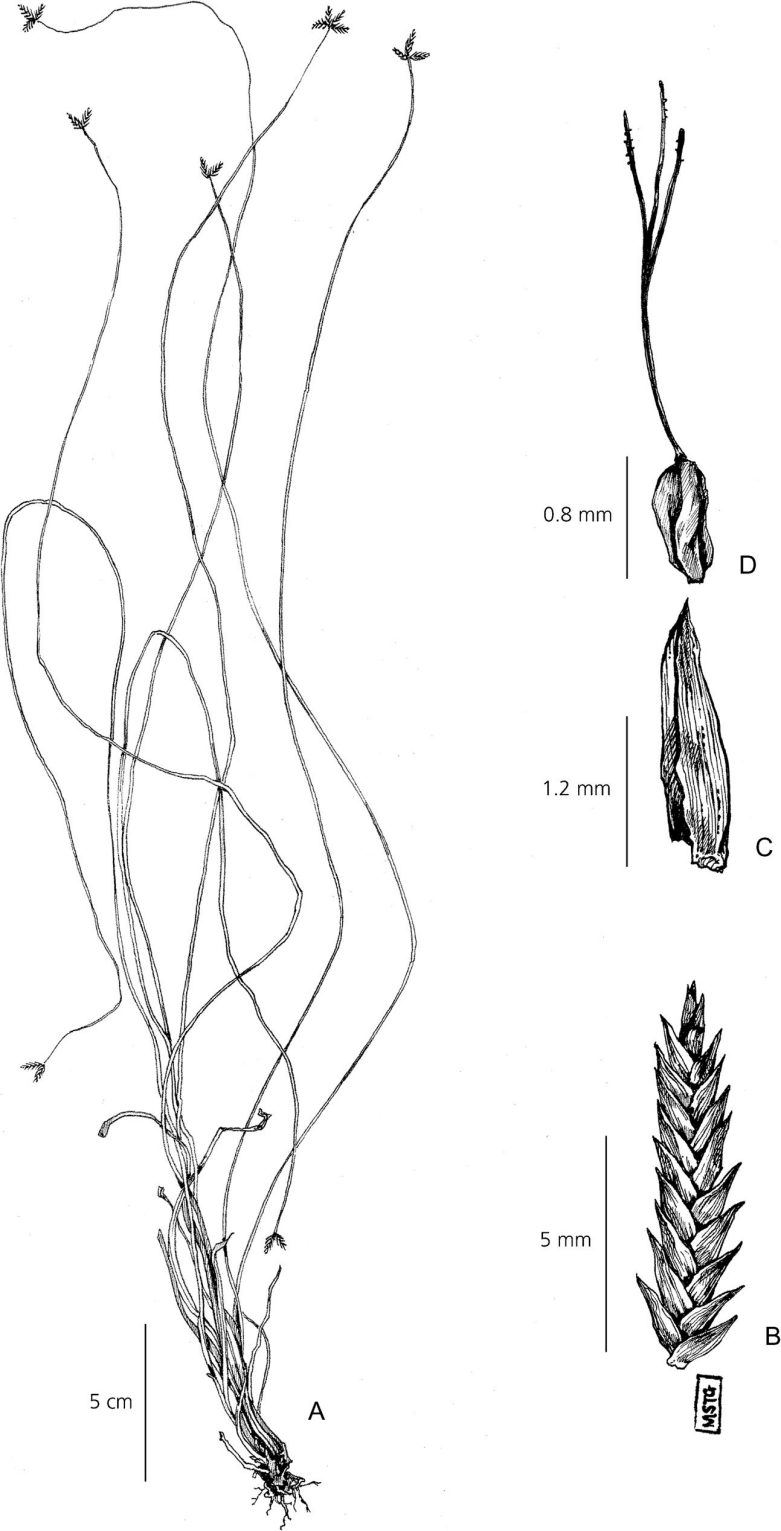
*Cyperus debilissimus*. **A** habit; **B** spikelet; **C** glume; **D** nutlet. **A** from *Baron* 3374; **B, C, D** from *Mesmer* 895. drawn by m. griffiths.

Perennial herb. *Rhizomes* short, tough. *Culms* densely tufted, long and slender, < 300 cm × 1 mm, flexuose, trigonous, densely cespitose, with up to 3 brown encircling sheaths, reaching up to 30 cm along the axis, aphyllous. *Involucral bracts* 2 – 4, short and inconspicuous. *Inflorescence* usually anthelate digitate and simple, but sometimes sessile or pedicellate. If present, 2 – 6 rays per inflorescence, rays can reach up to 3 cm long, and bear 4 – 6 spikelets. *Spikelets* lanceolate-linear, narrowly imbricate, strongly distichous, 3 – 10 × 1 – 2 mm, up to 30 flowers per spikelets, rachilla minutely winged. Spikelets can become viviparous and take root at maturity. *Glumes* lancolate, 1.5 – 2 × 1 mm, reddish-brown, darker towards pointed apex. *Stamens* 3, anthers smooth, yellow to white, setulose at apex. *Stigmas* 3, 3 mm long, far exceeding the glumes. *Nutlet* small, light brown, 0.4 – 0.8 × 0.3 mm wide, strongly trigonous with narrowly truncated base, rugolose surface. Fig. 7.

**distribution**. *Cyperus debilissimus* is endemic to the mountains of Central-Eastern and south-eastern Madagascar. Collections for the species have been taken from the provinces of Fianarantsoa, Antananarivo, and Toamasina. All herbarium vouchers for this species indicate that it occurs at high altitudes, between 1200 – 2300 m.

**specimens examined.** **madagascar**: central Madagascar, Nov. 1885, *Baron* 3374 (K); Fianarantsoa, Parc National d’Andringitra, Foret de Ravaro, 12.5 km SW of Antanitotsy, [22°12.7'S, 46°50.7'E], 22 Feb. 2002, *Mesmer* 895 (K); Massif du Manongarivo, April 1921, *Perrier de la Bathie* 13739; Massif de L'Andohahelo, 1889 m, 21 Oct. 1928, *Humbert* 6174 (US).

**habitat**. This species has been found on the siliceous rock ridges of the South-Central-Eastern Madagascan massifs, as well as the shady, damp undergrowth of tropical montane forests, and recently burnt areas.

**conservation status**. *Cyperus debilissimus* is only known from five georeferenced locations across Madagascar, resulting in a small estimated EOO of 9398 km^2^ and AOO of 24 km^2^ (below the threshold for Endangered EN B2). Three of the five locations where this species has been found border heavily degraded agricultural land. These populations are consequently at risk of destruction by expansion of arable land by slash-and-burn agriculture, as well as charcoal production and disrupted annual burning cycles. For these reasons, we assess *C. debilissimus* as Endangered EN B2ab(i,ii).

**notes**. Chermezon (1937) noted that two morphotypes of this species exists. The juvenile form is leafy and displays a scabrid culm and numerous well-developed leaf blades, with distinct, leafy involucral bracts. The mature form is aphyllous, with heavily reduced involucral bracts and a smooth, elongated stem which can reach 300 cm in length. These elongated stems grow through and hang from vegetation of the forest understorey. This dimorphism can be observed after burning cycles, where the leafy form of the plant regrows from the rhizomes which remain after the aphyllous form has been burnt.

**Cyperus fertilis** *Boeckeler* (1883: 90). Type: Sierra Leone, Mungo, 1874, *O. Boeckeler* s.n. (holotype B! [B_10_0278327], verified by Beentje 2009).

*Cyperus lanceola* Ridl. (Ridley 1884: 134). Type: Angola, May 1856, *F. M. J. Welwitsch* 7094 (lectotype **designated here**: B! [M000922448]; isolectotype: BM000922447).

**Fig. 8:**
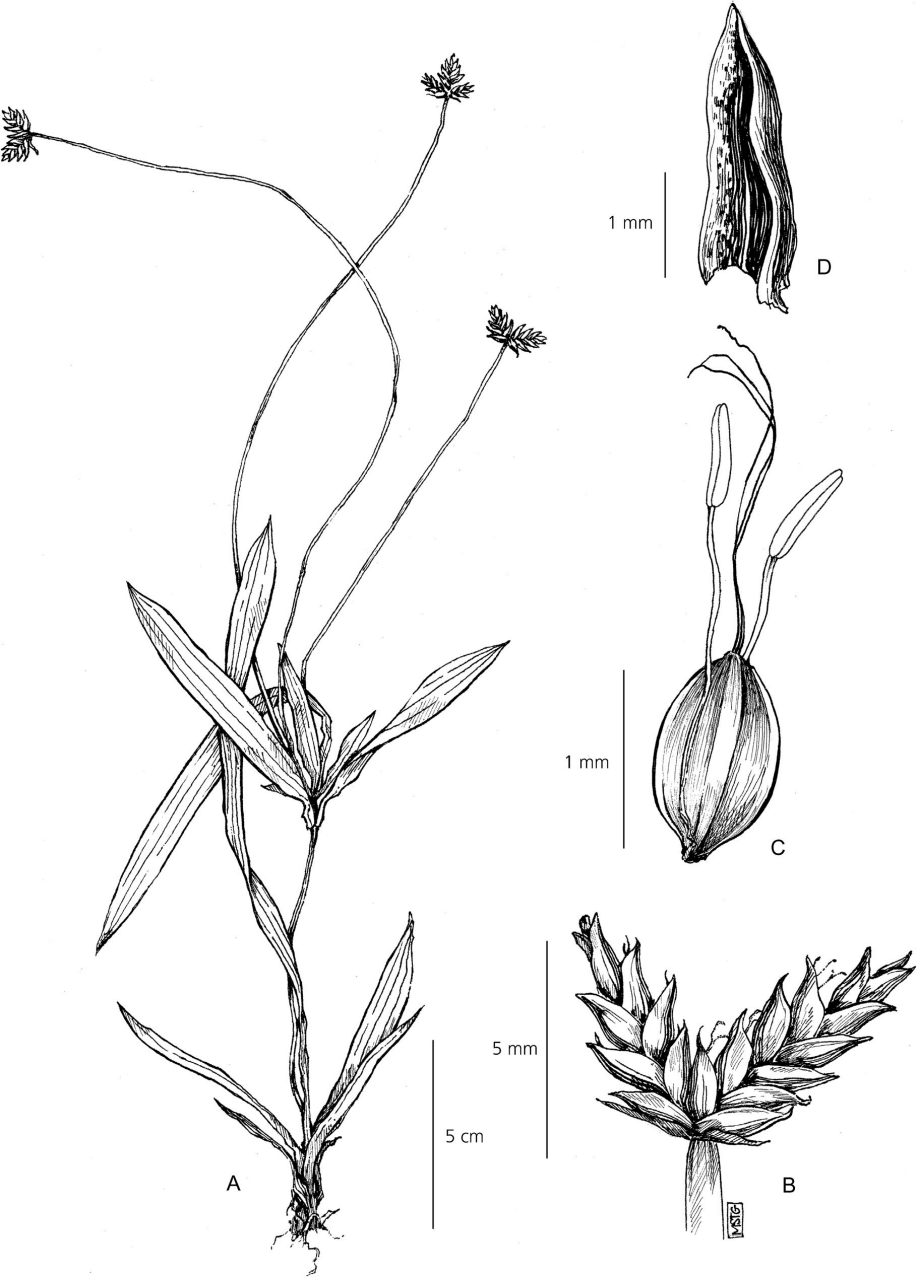
*Cyperus fertilis*. **A** habit; **B** spikelet; **C** glume; **D** nutlet. **A** from *Jones & Onochie* 17245, **B** from *Van der Veken* 8940, **C** and **D** from *Leeuwenberg* 9830. drawn by m. griffiths.

Herbaceous, tufted annual, sometimes perennial, often showing proliferous growth. *Rhizomes* short. *Culms* short, usually less than 150 cm × 1 – 3 mm, triquetrous or flat, smooth. *Leaves* lanceolate to ellipsoid, subtending the culm in a rosette, c. 10 – 17 cm × c. 10 – 13 mm, with white or purple venation, purple leaf sheath. *Involucral bracts* leaf-like, 4 – 9, with the shortest being 8 – 13 cm × 6 – 18 mm, spreading outwards. *Inflorescence* simple, anthelate, with spikelets atop 6 – 9 long, flexuous, stem-like rays, 20 – 30 cm long. Each ray bears a cluster of 1 – 3 spikelets. *Spikelet*s 3 – 5 × 2 – 3 mm, ovate, flattened, bearing 8 – 12 flowers. *Glumes* lanceolate 2.5 – 3 × 2 mm, with an acute apex, white to green at the keel, set at 45° to the rachilla, 3 – 4-nerved with an acute apex. *Stamens* 3, anther linear and smooth, 1.8 mm long. *Stigmas* 3, 2.8 – 3.2 mm long, exceeding the glumes. ***Nutlet*** 1.8 – 2 × 1 mm, trigonous, ellipsoid with truncated base, brown and smooth. Fig. 8.

**distribution**. *Cyperus fertilis* is distributed widely across central and western Africa from Cameroon, Central African Republic, Congo, Equatorial Guinea, Gabon and Ghana, to the Ivory Coast, Liberia, Nigeria and Zaire (Fig. 4). It is also native to Angola and Northern Madagascar.

**specimens examined.** **angola**: Cabinda; Belize, on the margins of the Luali R., 12 Aug. 1918, *Gossweiler* 7610 (COI); on the stream joining the Canguerasange river, 10.1854, *Welwitsch* 7094 (BM). Cameroon, 42 km S of Kumba, on the outskirts of Mt Cameroon National Park, 50 m, [04°16'48" N, 09°17'24" E], 4 May 1972, *Leeuwenberg* 9830 (WAG). Democratic Republic of the Congo, Kivoe, Irangi, beside the Luhoho R., 800 – 850 m, *Van der Veken* 8940 (G). Sierra Leone, Mungo, Sept. 1874, *Boeckeler* s.n. (B). Gabon, Moyen-Ogooue, 55 m, [00°43'35" S, 10°33'27" E], 10 Feb. 2012, *Stevart & Droissart* 4215 (MO).

**habitat**. Like other species in sect. *Incurvi*, *Cyperus fertilis* occurs in the damp undergrowth of tropical forests, including riverbanks and disturbed areas, such as the margins of tracks and clearings.

**conservation status**. The wide distribution across Africa results in an EOO of 8,558,519.669 km^2^. This range is above the threshold necessary to classify a species as Threatened (EOO >20,000 km^2^, AOO >2000 km^2^). Considering the size, health and geographic range of these populations across Africa, *Cyperus fertilis* is not believed to be a priority for conservation action at this time. For these reasons, *C. fertilis* is here assessed as Least Concern LC.

**notes**. The species is able to spread via proliferous growth, whereby adventitious buds that form on leaves and flowers are capable of rooting and developing into new plants.

**Cyperus mapanioides** *C.B.Clarke* (1894: 568; 1901: 340). Type: Democratic Republic of the Congo, Stanley pool, 304 m, 23 July 1888, *Hens* 69 (lectotype **designated here**: L! [L0042414]; isolectotype: BR! [BR0000008644763]).

*Cyperus dichromeniformis* var. *Major* Boeckeler, *Flora* 62: 549 (1879) ≡ *Cyperus major* (Boeckeler) Cherm. (Chermezon 1922: 29) ≡ *Cyperus mapanioides* var. *major* (Boeckeler) Kük. (Kükenthal 1935 – 1936: 230). Type: Central African Republic, 31 May 1871*, Schweinfurth* 3461 (lectotype **designated here**: K! [K000321331]).

*Cyperus major* var. *micranthus* Cherm. (Chermezon 1935: 282) ≡ *Cyperus mapanioides* f. *micranthus* (Cherm.) Kük. (Kükenthal 1935 – 1936: 231). Type: Democratic Republic of the Congo, July 1932, *H. Vanderyst* 33158 (lectotype **designated here**: BR! [BR0000009887619]).

**Fig. 9:**
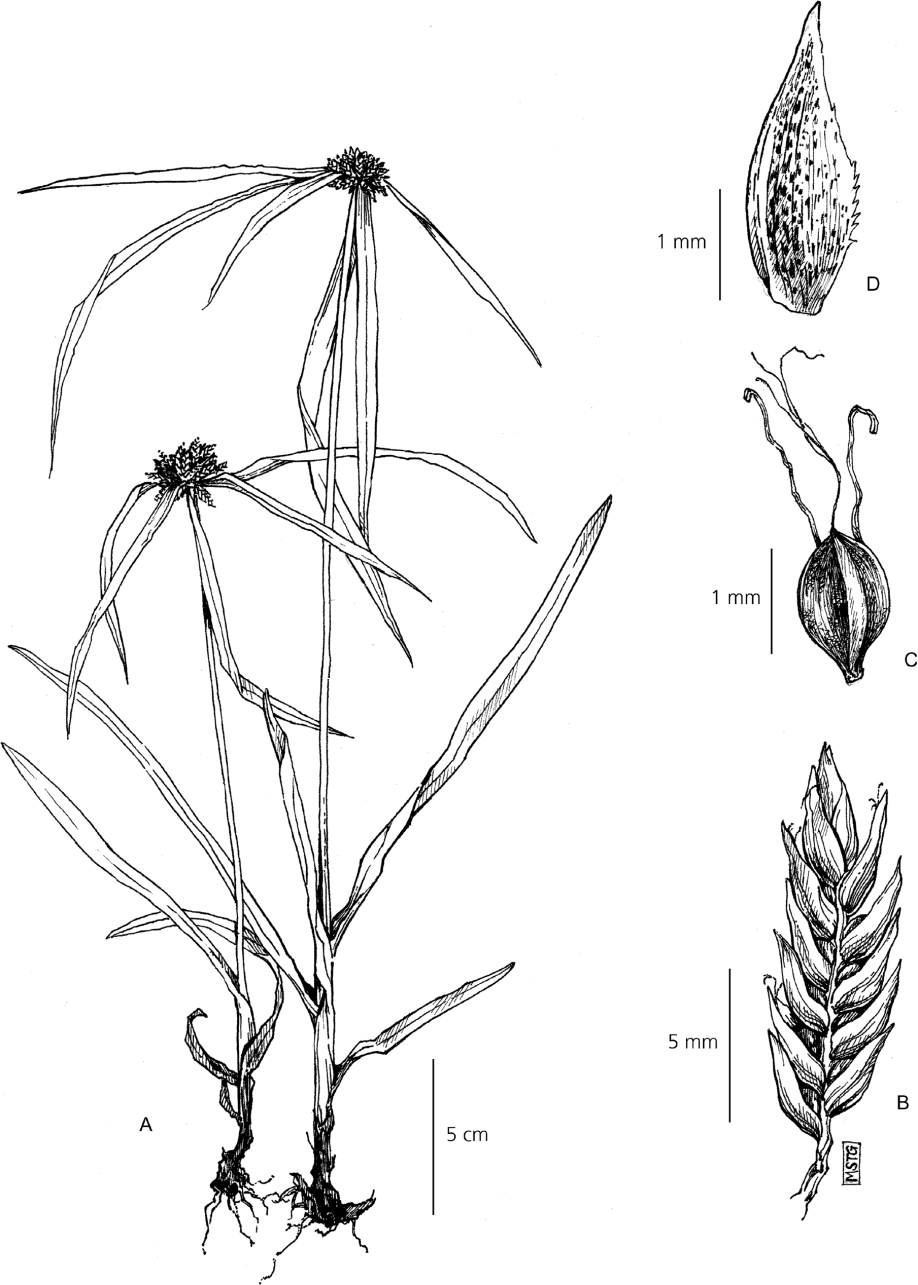
*Cyperus mapanioides*. **A** habit; **B** spikelet; **C** lateral view of glume; **D** nutlet. **A** from *Haba* 418; **B, C, D** from *Denys* 1035. drawn by m. griffiths.

Herbaceous perennial. *Rhizomes* fibrous, tangled, creeping, with small, scaly stolons. *Culms* rigid, 15 – 50 cm × 1.4 – 3.9 mm, trigonous to triquetrous, glabrous. *Leaves* up to 40 × 0.4 – 1.2 cm; leaf blade linear, scabrid at margins, subtle 3-nerved venation, apex acute; leaf sheath 1.5 – 7 cm long, transitioning from reddish-brown to deep purple at the base. *Involucral bracts* 4 – 7, leaf-like, 10 – 34 × 0.6 – 1.3 cm, spreading. *Inflorescence* a single loosely capitate cluster of pale brown, sessile spikelets, 8 – 16 spikelets per inflorescence. *Spikelets* linear-lanceolate to ovoid, 7 – 18 × 2.4 – 4 mm, bearing 8 – 16 flowers, rachilla winged*. Glumes* 2.5 – 3 × 2 mm, lanceolate-ovate, whitish-grey and veined, scabrid at the margins, spreading when mature, apex acute*. Stamens* 3; anther 1.3 – 3 mm long. *Style* 3-branched, 0.6 – 1.3 mm long. *Nutlet* ellipsoid-obovoid, trigonous, 1.4 – 1.9 × 0.9 – 1.3 mm, brown, smooth, sometimes minutely papillose. Fig. 9.

**distribution**. *Cyperus mapanioides* is widely distributed across tropical West, tropical Eastern and central Africa, and can be found as far south as Angola, Zambia, and northern Madagascar (Fig. 4).

**specimens examined.** **democratic republic of the congo**: Wapinda, [3°37'N, 22°44'E], 2 Aug. 1984, *Denys* 1035 (GENT); Stanley pool, 304 m, 23 July 1888, *Hens* 69 (BR); Stanley Pool, 100 m, 12 April 1894, *Hens* 7B (BR); Bas-Congo, Boma, 100 m, 12 Dec. 1889, *Hens* 389 (L); Plateau des Bateke net de Aumi, 2 Feb. 1972, *Robbrecht* 1720 (BR); Katanga, Kyamasumba, 100 m, [27°09'00"E, 10°13'48"S], *Malaisse & Robbrecht* 2283 (BR). **republic of guinea**: Guinea Forest, Simandou Range, on the track running between Canga and Whiskey, 981 m, [08°33'16"N, 08°53'08"W], 18 Nov. 2008, *Haba* 418 (K).

**habitat**. *Cyperus mapanioides* is an understorey plant found primarily among the leaf litter of the damp forest floor, as well as in disturbed areas such as clearings and along footpaths. The species has been noted to occur in the Miombo forests of central and southern Africa, which are expanses of grasslands, savannas and shrublands characterised by the dominance of Miombo trees (*Brachystegia* spp.).

**conservation status**. The wide distribution of this species across Africa has amplified its estimated EOO to 8,144,374 km^2^. This range is above the threshold necessary to classify a species as Threatened (EOO >20,000 km^2^). In light of the size, health and range of this species across Africa, *Cyperus mapanioides* is not considered a conservation priority at this time, and is assessed as Least Concern LC.

**Cyperus molliglumis** *Cherm*. (Chermezon 1925b: 615). Type: Madagascar, Forêt d'Andasibé, *H. Perrier de la Bâthie* 17159 (lectotype **designated here**: P00450808, isolectotype: K000362672).

**Fig. 10:**
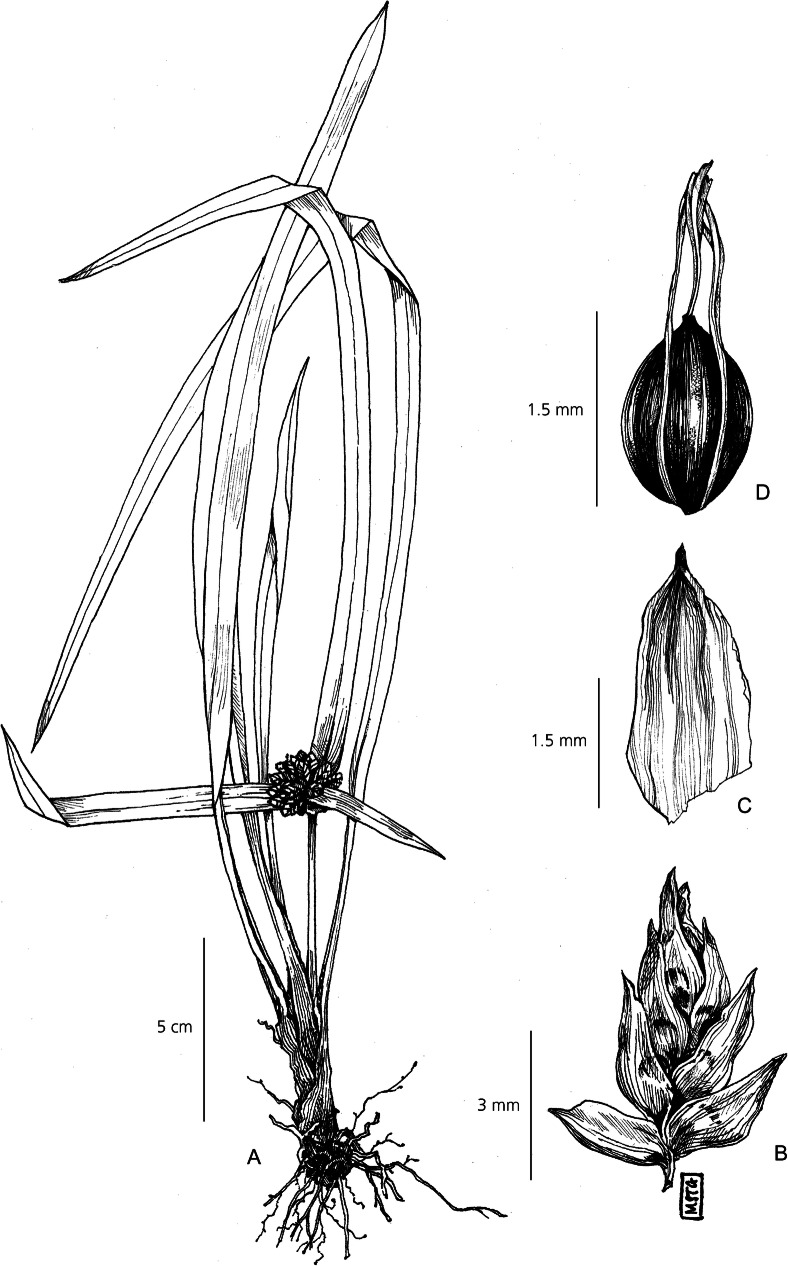
*Cyperus molliglumis*. **A** habit; **B** spikelet; **C** glume; **D** nutlet. All from *Perrier de la Bâthie, H.* 17159. drawn by m. griffiths.

Herbaceous perennial. *Rhizomes* short, woody. *Culms* rigid, 5 – 15 cm × 1 – 2 mm, trigonous and smooth, within basal rosette of many leaves. *Leaves* long, narrowly lanceolate, 20 – 40 × 1 – 1.5 cm, far exceeding the height of the culm, subtle 3-nerved venation running the length of the leaf, leaf margins fold abruptly towards the base, drawing the venation into a sheathed pseudopetiole 6 – 8 cm × 4 mm, transitioning from green to purple at the base. *Involucral bracts* leaf-like, 3, variable in length with typically one long, one medium length and one short, 5 – 20 × 1.5 – 2 cm. *Inflorescence* a simple, densely capitate cluster of many sessile spikelets, 1.2 cm wide. *Spikelet*s ovate-lanceolate and obtuse, 5 – 9 × 3 mm, 8 – 16 flowers per rachilla. *Glumes* densely imbricate, spreading when mature, ovate and strongly mucronate, 3 × 2 mm, straw yellow with green margins. *Stamens* 3, anthers linear, tip smooth. *Stigmas* deeply 3-branched, 1 mm long. *Nutlet* ellipsoid, obtusely trigonous, 1.2 – 1.5 × 0.8 – 1 mm, minutely papillose, dark reddish-brown. Fig. 10.

**distribution**. *Cyperus molliglumis* is endemic to the eastern escarpment of Madagascar, and has been collected from the provinces of Antsiranana, Fianarantsoa, and Toliara, at altitudes between 400 – 1500 m.

**specimens examined.** **madagascar**: Forêt d'Andasibé, 900 m, *Perrier de la Bâthie,* 17159 (K); Forêt d'Andasibé, 900 m, *Perrier de la Bathie* 17162 (P); Toliara, Bemangidy, 420 m, [24°33'45"S, 47°12'18"E], 10 Dec. 2011, *Gautier* 5781 (G).

**habitat**. This species primarily occurs in the humid understorey of tropical montane forests and shrublands, but has also been found on stream margins and rocky slopes.

**conservation status**. The extended distribution of *Cyperus molliglumis* across Madagascar has amplified its EOO to 2346 km^2^, while the minimal AOO for the species remains low, at 12 km^2^ (below the threshold for EN B1,2a). Habitat degradation due to slash-and-burn agriculture, and urban development are the primary threats to *C. molliglumis*. All collection localities for this species occur in the evergreen forests of the eastern escarpment, an ecoregion suffering from reductions in both the area and the quality of forest cover; inferred from satellite imagery and primary vegetation maps (Du Puy & Moat 1996). Consequently, we predict a reduction in both the EOO and AOO of *C. molliglumis*, which is assessed as Endangered EN B1ab(i,ii,iii)+2ab(i,ii,iii).

**Cyperus multinervatus** *Bosser* (1955: 119). Type: Madagascar, Antsiranana, Andapa, Lokoho Basin, Tributary of the Ankasahana, 450 – 500 m, 1948, *Humbert* 22011 (lectotype **designated here**: P! [P00450811]; isolectotype: P! [P00580645]).

Herbaceous perennial. *Rhizomes* short. *Culms* short, 3 – 6 × 0.2 cm long, acutely trigonous, enclosed within basal tuft of leaves. *Leaves* far exceeding the culm, up to 75 cm × 10 – 20 mm wide, linear to lanceolate, with prominent 3-nerved venation running the length of the blade, margin scabrid. Leaf blade folds basally to create a channelled pseudopetiole just above the leaf sheath. *Pseudopetiole* 3 – 5 cm long, transitioning to purplish-red at the base, vertical purple striations running its length. *Involucral bracts* 3 – 4, leaf-like, 10 – 60 cm × 6 – 20 mm. *Inflorescence* simple, condensed-capitate, 1.5 – 2 × 1.5 – 2 cm, many spikelets. *Spikelets* erect, strongly linear-lanceolate 15 – 18 × 1 – 2 mm, bearing 10 – 20 flowers per rachis. *Glumes* large, 5 – 6 × 2 – 3 mm, densely imbricate, broadly ovate, briefly mucronate at the apex, smooth margins with white vertical striations. *Stamens* 3, anthers linear, 1 mm long, smooth. *Style* strongly three-branched. *Nutlet* ellipsoid, obtusely trigonous.

**distribution**. This species is only known from a single location in Marojejy National Park, in the forests of north-eastern Madagascar.

**specimens examined.** **madagascar**: Andapa, SAVA, the basin of the Lokoho (NE), 450 – 550 m, Dec. 1948, *Humbert* 22011 (P! [P00450811], P! [P00580645]).

**habitat**. Collected near a tributary for the Ankasahana river, likely a forest understory plant.

**conservation status**. Due to the pronounced lack of information surrounding the distribution, population size, habitat preferences and life history of this species, we assess *Cyperus multinervatus* as Data Deficient at this time.

**notes**. Easily confusable with *Cyperus molliglumis*. Distinguishable by its short culm, distinctive erect spikelets which are strongly linear-lanceolate, and large glumes.

**Cyperus pandanophyllum** *C.B.Clarke* (1908: 8). Type: Madagascar, 1833, *Goudot* s.n. (holotype: G! [G00018589]; isotype: P00450828).

**Fig. 11:**
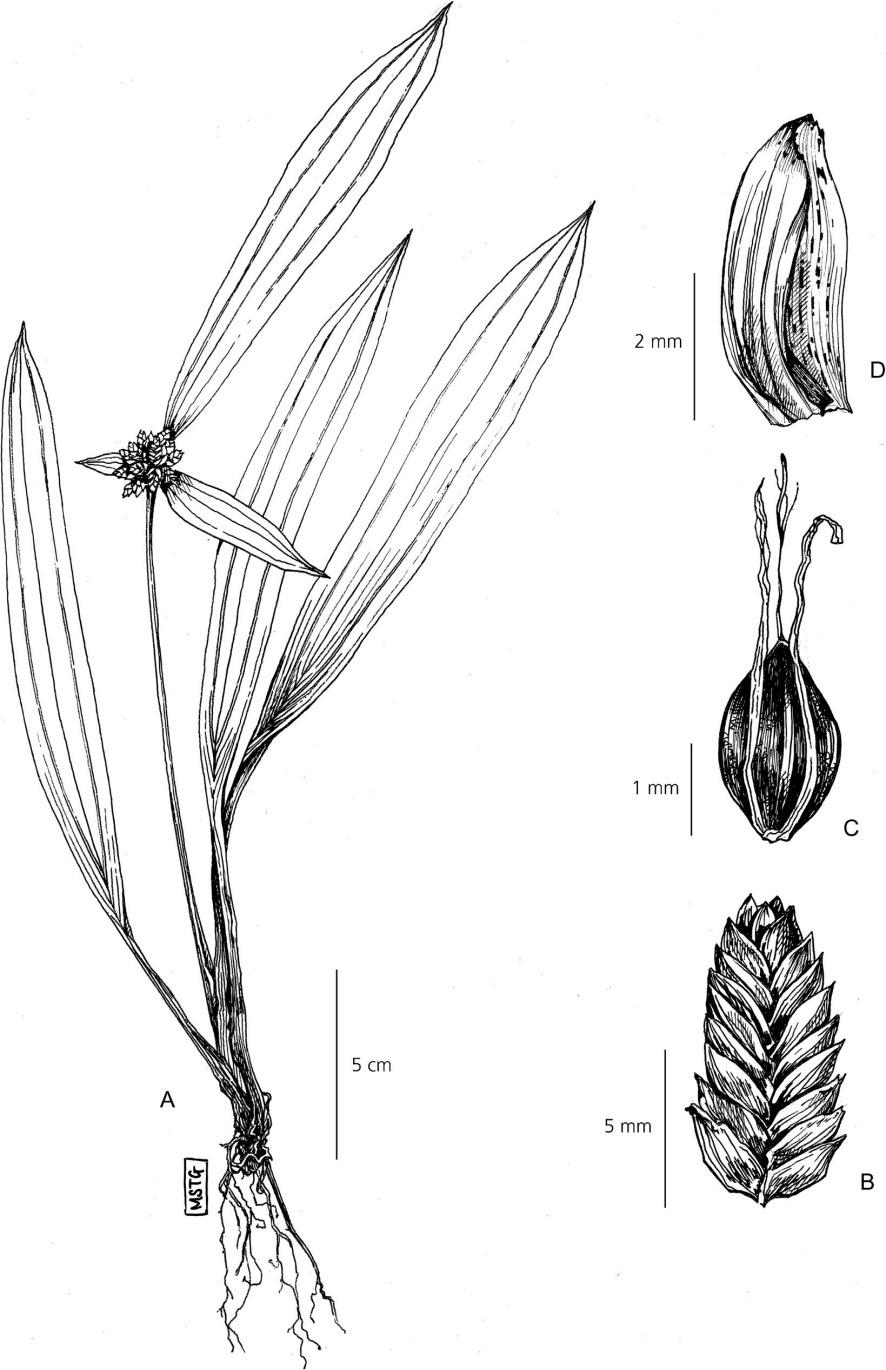
*Cyperus pandanophyllum*. **A** habit; **B** spikelet; **C** nutlet; **D** glume. **A** from *Goudot* s.n. **B, C, D** *Beentje* 4773. drawn by m. griffiths.

Herbaceous perennial. *Rhizomes* short and oblique. *Culms* 15 – 25 cm × 1.5 – 5 mm, strongly trigonous to the extent of being 3-winged, flattened towards the base, bearing many leaves. *Leaves* lanceolate-ovate, up to 20 × 1.5 – 4 cm, far exceeding the culm, 3 prominent veins running the length of the blade. Leaf margins folding basally to produce a sheathed pseudopetiole, 5 – 10 cm × 5 – 10 mm, tapering and turning purple towards the base. *Involucral bracts* leaf-like, 3, lanceolate, variable in length – typically one long, one medium length and one short, up to 20 × 1.5 – 2 cm, far exceeding the inflorescence. *Inflorescence* 1 – 1.5 × 1.5 – 2 cm simple-capitate cluster of many sessile spikelets. *Spikelet*s lanceolate-ovate, 6 – 10 × 3 – 5 mm, rachilla minutely winged, 16 – 25 flowers per rachilla. *Glumes* densely imbricate, 3 – 4 × 2 mm, soft green and shortly mucronate, with many vertical striations. *Stamens* 3, anther tip smooth. Stigma 3-branched, style 1.5 mm long. *Nutlet* obtuse, 2 × 2 mm, trigonous, dark reddish-brown, minutely rugolose. Fig. 11.

**distribution**. *Cyperus pandanophyllum* is endemic to the forests of eastern and north-eastern Madagascar.

**specimens examined.** **madagascar**: Moramanga, Mantady National Park, 995 m, [18°50'S, 48°30'E], 29 Dec. 1992, *Beentje* 4774 (K); Vakinankaratra, 1290 m, [46°42'22"S, 19°22'24"E], 1833, *Goudot* s.n (P).; l’Onive el du Mangoro, 200 m, Feb. 1925, *Perrier de la Bathie* 12161 (K).

**habitat**. This species has been collected primarily from the dark undergrowth of the humid eastern forests. It has also been found in highly disturbed forest areas, along rivers, and in freshwater wetlands. All collections for this species have been made from the province of Toamasina.

**conservation status**. *Cyperus pandanophyllum* has a low minimum AOO of 24 km^2^ (meeting the criteria for Endangered EN B2), but the expansive range of its distribution across Toamasina has meant that the estimated Extent of Occurrence (EOO) is fairly high, at 37,370 km^2^. Despite this, the species is only known from four locations, meaning that the species is highly vulnerable to future threats or stochastic environmental changes (meeting the criteria for EN B2a). With the eastern evergreen forests being lost at an estimated rate of 1 – 2% annually (Vieilledent *et al.* 2018), we predict that the primary threats to *C. pandanophyllum*, forest exploitation by subsistence farmers, illegal logging and charcoal production, are likely to further restrict the AOO and EOO. For these reasons, we assess *C. pandanophyllum* as Endangered EN B2ab(i,ii,iii).

**Cyperus plantaginifolius** *Cherm*. (Chermezon 1919, publ. 1920: 346). Type: Madagascar, Forêt d'Analamazaotra, 900 m, *Perrier de la Bâthie, H.* 6340 (lectotype **designated here**: P! [P00450845], isolectotype: P00450844).

*Cyperus plantaginifolius* var. *minor* Cherm. (Chermezon 1925b: 615). Type: Madagascar, Forêt d'Andasibé, 900 m, Feb. 1925, *Perrier de la Bâthie, H.* 17158 (holotype: P! [P00450847], isotype: P! [P00450849]).

**Fig. 12:**
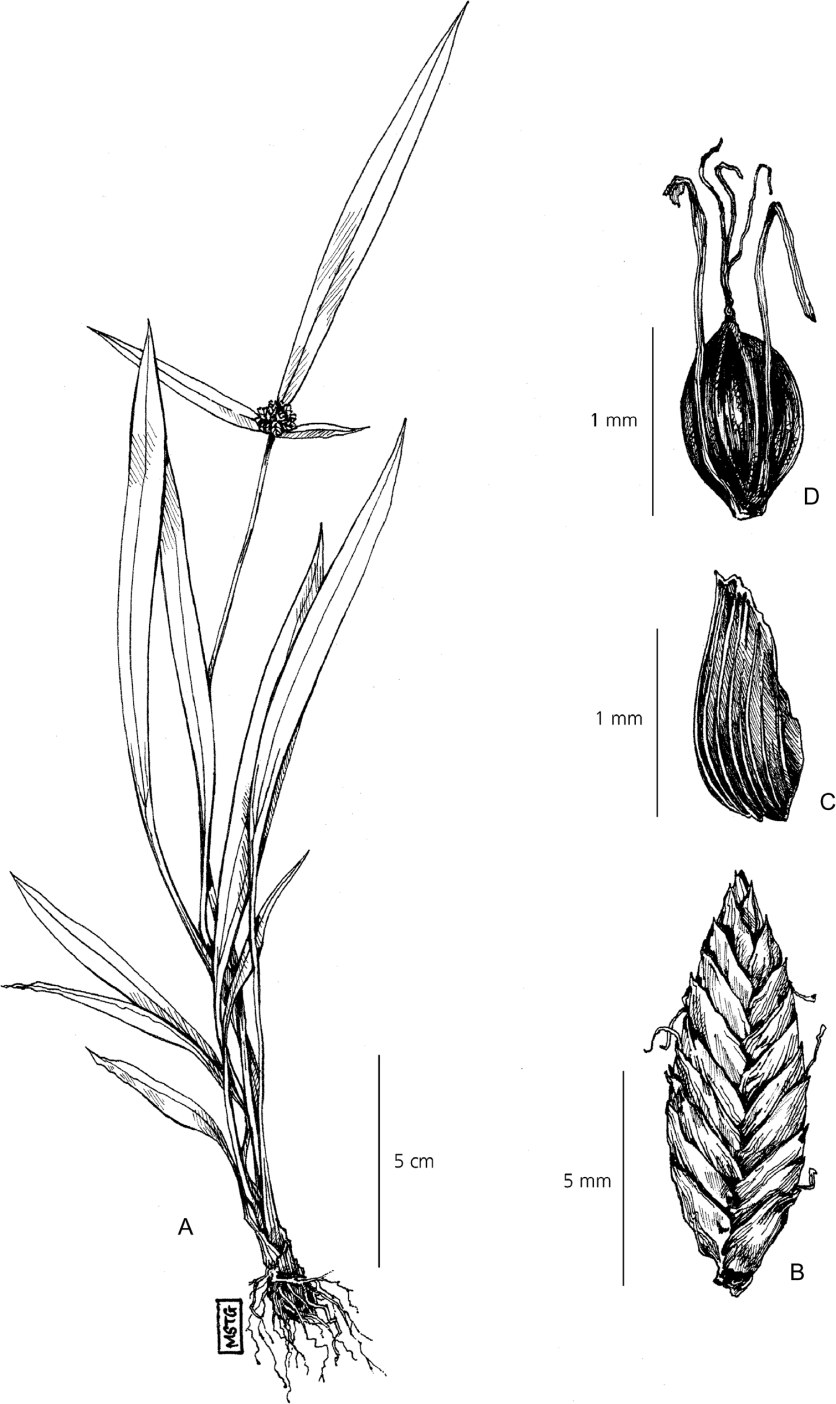
*Cyperus plantaginifolius*. **A** habit; **B** spikelet; **C** glume; **D** nutlet. **A** from *Leandri* 1586; **B, C, D** from *Ralimanana* 1418. drawn by m. griffiths.

Herbaceous perennial. *Rhizomes* short and woody. *Culms* rigid, 15 – 45 cm × 1.4 – 2 mm, trigonous and smooth, enclosed within the sheaths of the leaves. *Leaves* narrowly lanceolate, 10 – 25 cm × 15 mm, scabrous on both abaxial and adaxial surface. Leaf margins fold towards the base to create a sheathed pseudopetiole, 8 – 15 cm × 2 – 4 mm, transitioning to purplish-red at the base. *Involucral bracts* leaf-like, 3, variable length – typically one short, one medium length and one long, up to 18 × 1.5 cm, spreading away from, and far exceeding, the inflorescence. *Inflorescence* simple, 0.5 – 1.5 × 1.5 – 2 cm narrowly capitate, sub-spherical clusters of 8 – 25 sessile spikelets. *Spikelet*s oblong-ovate, 5 – 10 × 3 – 4 mm, 16 – 28 flowers per rachilla. *Glumes* densely imbricate, oval-obtuse, 1 – 3 × 1 – 1.2 mm, briefly mucronate, pale-straw coloured with a paper-like texture, multinerved with smooth edges. *Stamens* 3, anther linear, tip smooth. *Stigmas* 3-branched, strongly exserted, 1.3 mm long. *Nutlet* widely ellipsoid, trigonous, truncated at base, dark reddish-brown, lightly papillose surface. Fig. 12.

**distribution**. *Cyperus plantaginifolius* is a perennial sedge endemic to the montane forests of central eastern Madagascar. This species is typically found on humid massifs at medium altitudes, between 500 – 1500 m.

**specimens examined.** **madagascar**: Sandrangato, South of Moramanga, 800 – 1000 m, 3 – 7 Nov. 1952, *Leandri* 1586 (P); Andasibe Forest, 900 m, Feb. 1925 *Perrier de la Bathie* 17158 (K); Antananarivo, Analamazoatra, 900 m, *Perrier de la Bathie* 6340 (P); Mandraka, Imerina Forest, Oct. 1905, *d'Alleizette* 502 (P); Toamasina, Moramanga, Ambohibary, Sahaivo forest, 1065 m, [18°50'29"S, 48°17'51"E] *Gereau* 2008 (MO); Toamasina, Ambatondrazaka, Tsiazomborona Forest, 1100 m, [18°02'58"S, 48°32'21"E], 20 Nov. 2005, *Haevermans & Ranaivo* 3566 (P); Mahajanga, Tsaratanana, Andranoampanga Forest, 1 h 30 minute walk from Tsarahonenana Village, [17°40'53"S, 48°00'06"E], 18 Jan. 2010, *Ralimanana* 1418 (K); Mahajanga, Tsaratanana, Andranonampanga Forest [17°40'53"S, 48°00'06"E], 18 Jan. 2010, *Rakotonasolo* RLI 1418 (TAN).

**habitat**. Like other rainforest-dwelling members of *Cyperus* sect. *Incurvi*, this species has been documented among the shaded leaf litter of the forest understorey, as well as on the rocky slopes of the central-eastern massifs.

**conservation status**. *Cyperus plantaginifolius* has a minimal AOO of 76 km^2^, and an estimated EOO of 75,933 km^2^ (meeting the threshold for Vulnerable VU B2). Within this range, the species falls into nine separate locations (qualifying for VU B2a), where primary threats, conversion of forests to agricultural land, conversion of wetlands to rice paddies, mining, and charcoal production, affect each independently (Phillipson *et al.* 2010; Bamford *et al.* 2017; Lammers *et al.* 2015). Of these nine locations, six are included within the protected area network, however; concerns over the efficacy of the protection within these areas, and the threat of intensifying deforestation in these areas in the wake of COVID-19, mean that the AOO, EOO and area, extent and/or quality of habitat of the species are projected to decline. For these reasons, we assess *C. plantaginifolius* as Vulnerable VU B2 ab(i,ii,iii).

**additional notes**. Easily confusable with the African species *Cyperus mapanioides*. Distinguishable by its lanceolate leaves, the presence of a pseudopetiole, and its shorter, non-ciliate glumes. There is also notable ambiguity between *C. plantaginifolius* and *C. pandanophyllum*, which share overlapping distribution ranges in eastern Madagascar. *Cyperus plantaginifolius* is discernible by its smooth, narrower leaves, wingless stems and reduced number of spikelets per rachilla.

**Cyperus rufostriatus** *C.B.Clarke. ex Cherm*. (Chermezon 1919, publ. 1920: 347). Type: Madagascar, Masoala, 300 m, Oct. 1921, *H. Perrier de la Bâthie* 2571 (lectotype **designated here**: P! [P00466119]; isolectotype: P! [P00450878]).

*Cyperus hylophilus* Cherm. (Chermezon 1925b: 616). Type: Madagascar, Confluent de l'Onive et du Mangoro, 700 m, Feb. 1925, *H. Perrier de la Bâthie* 12152 (holotype: P00450573).

**Fig. 13:**
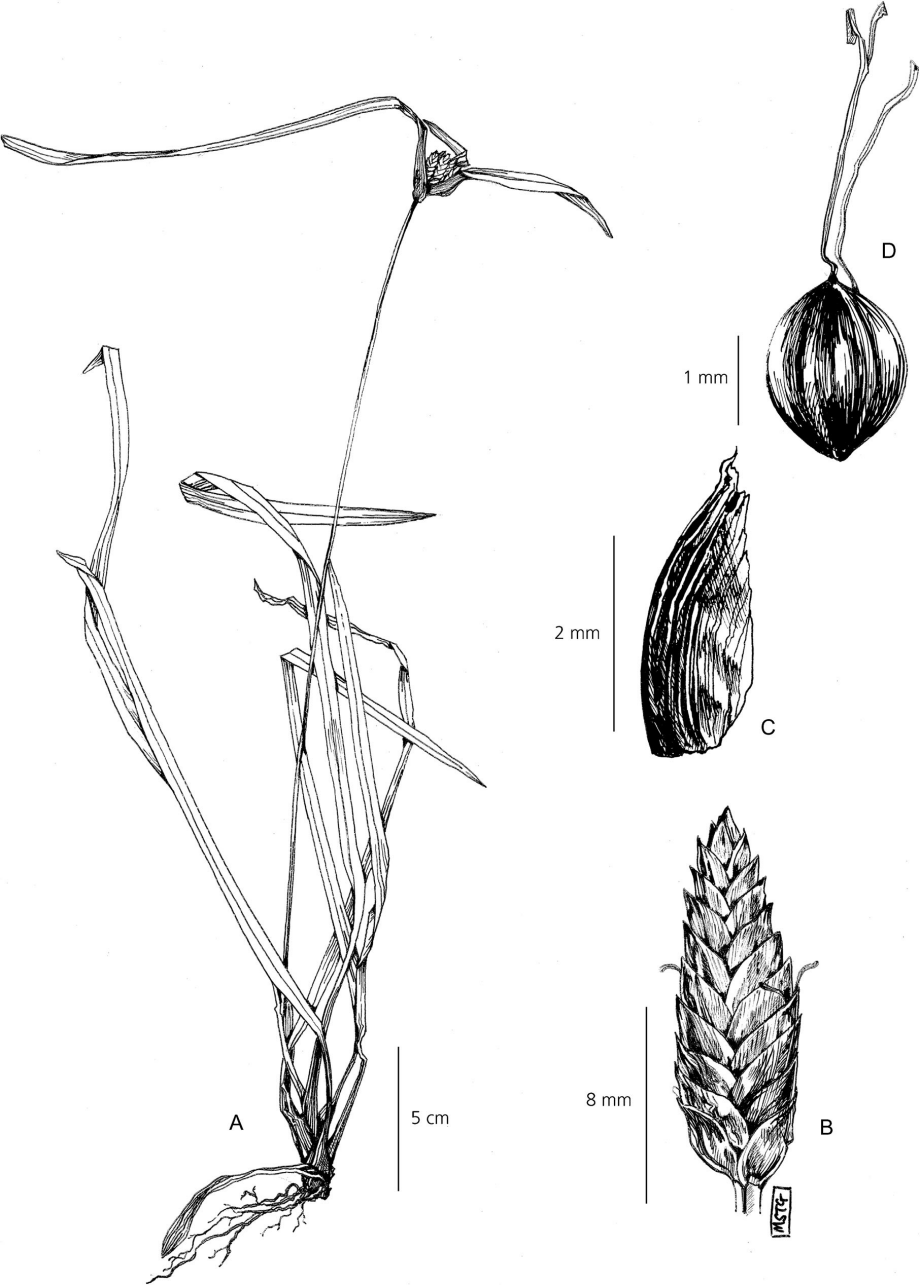
*Cyperus rufostriatus*. **A** habit; **B** spikelet; **C** glume; **D** nutlet. **A** from *Perrier de la Bâthie* 2571; **B, C, D** from *Perrier de la Bâthie* 12152. drawn by m. griffiths.

Loosely tufted perennial herb. *Rhizomes* short, emitting slender stolons. *Culms* slender, erect, 5 – 25 cm × 1 – 3 mm, strongly triquetrous to the extent of bearing 3 wings, often transitioning to red towards the base. *Leaves* linear to linear-oblong, 10 – 40 × 0.5 – 1.5 cm, 3 prominent veins run the length of the leaf blade, leaf narrows and folds at the base to create a channelled pseudopetiole 3 – 8 × 2 – 5 cm, mid-brown to dark reddish-purple towards the base. *Involucral bracts* leaf-like, 3 – 5, variable length up to 40 × 1.5 cm, spreading away from, and far exceeding, the inflorescence. *Inflorescence* simple capitate, 1 – 1.5 × 0.5 – 3 cm bearing 3 – 16 sessile spikelets. *Spikelet*s lanceolate and flattened with acute apices, 6 – 16 × 3 – 4 mm, strongly distichous, partially obscured by the subtending bracts, 10 – 14 flowers per rachilla. *Glumes* densely imbricate, ovate-subacute mucronate, 4 – 6 × 2 – 3 mm wide, medium green to dark straw yellow at the margins, with striations running the length of the glume, glabrous. *Stamens* 3, anthers linear, tip smooth. Stigma 3-branched. *Nutlet* trigonous, widely ellipsoid, 1.5 – 2 × 1.5 – 2 mm, surface lightly papillose. Fig. 13.

**distribution**. *Cyperus rufostriatus* is native to the eastern and north eastern forests of Madagascar. Collections of this species have been made in the Alaotra-Mangoro, and Analanjirofo regions of Toamasina, as well as the northerly Sava region of Antsiranana, at altitudes up to 1200 m.

**specimens examined.** **madagascar**: Masoala, 300 m, Oct. 1912, *Perrier de la Bathie* 2571 (P); Toamasina, Alaotra-Mangoro, Onive R., 700 m, [19°40'S, 47°54'E], Feb. 1925, *Perrier de la Bathie* 12152 (K); *Baron* 3254 (K); Antsiranana, SAVA, Marojejy, Ambatosoratra, 700 – 900 m, [14°32'00"S, 49°41'00"E], 24 Feb. 1989, *Miller* 4223 (K); Tamatave, W coast of Masoala Peninsula, Antalavia, 46 km SE of Maroantsetra, along trail beside the river running NE-SW, just South of the village, 19 Feb. 1988, *Simpson* 88/42 (K).

**habitat**. This species typically occurs in the understorey of humid montane forests, and has been recorded growing on steep slopes, and bordering streams, rivers, and swamps.

**conservation status**. The extensive geographic separation between the populations of *Cyperus rufostriatus* has inflated the estimated EOO to 31,056 km^2^, while the minimal AOO remains low, at 24 km^2^, reflecting the scarcity of collections made for this species (qualifying *C. rufostriatus* for Endangered B2, as AOO < 500 km^2^). Members of this species have been collected from five locations, where the primary threats to the species, deforestation by slash-and-burn agriculture, forest exploitation (illegal logging, mining and poaching) and charcoal production will impact them independently. Surviving in a restricted number of locations means that *C. rufostriatus* will be vulnerable to stochastic environmental changes, or to any intensification of the threats that it already faces (meeting the criteria for EN B2a, as number of locations = 5). Considering the projected lengthening of dry periods in the country as a result of climate change, on top of the devastation the Malagasy economy will face in the wake of COVID-19, we predict many of the threats to *C. rufostriatus* will intensify in the near future (Desbureaux & Damania 2018). Consequently, we project that the AOO, EOO and/or quality of habitat of *C. rufostriatus* are likely to decline, and so we assess this species as Endangered EN B2ab(i,ii,iii).

**Cyperus sciaphilus** *Cherm.* (Chermezon 1919, publ. 1920: 346). Type: Madagascar, Rivière Mananara, 200 m, Oct. 1912, *H. Perrier de la Bâthie* 2512 (lectotype **designated here**: P ! [P00450880]).

**Fig. 14:**
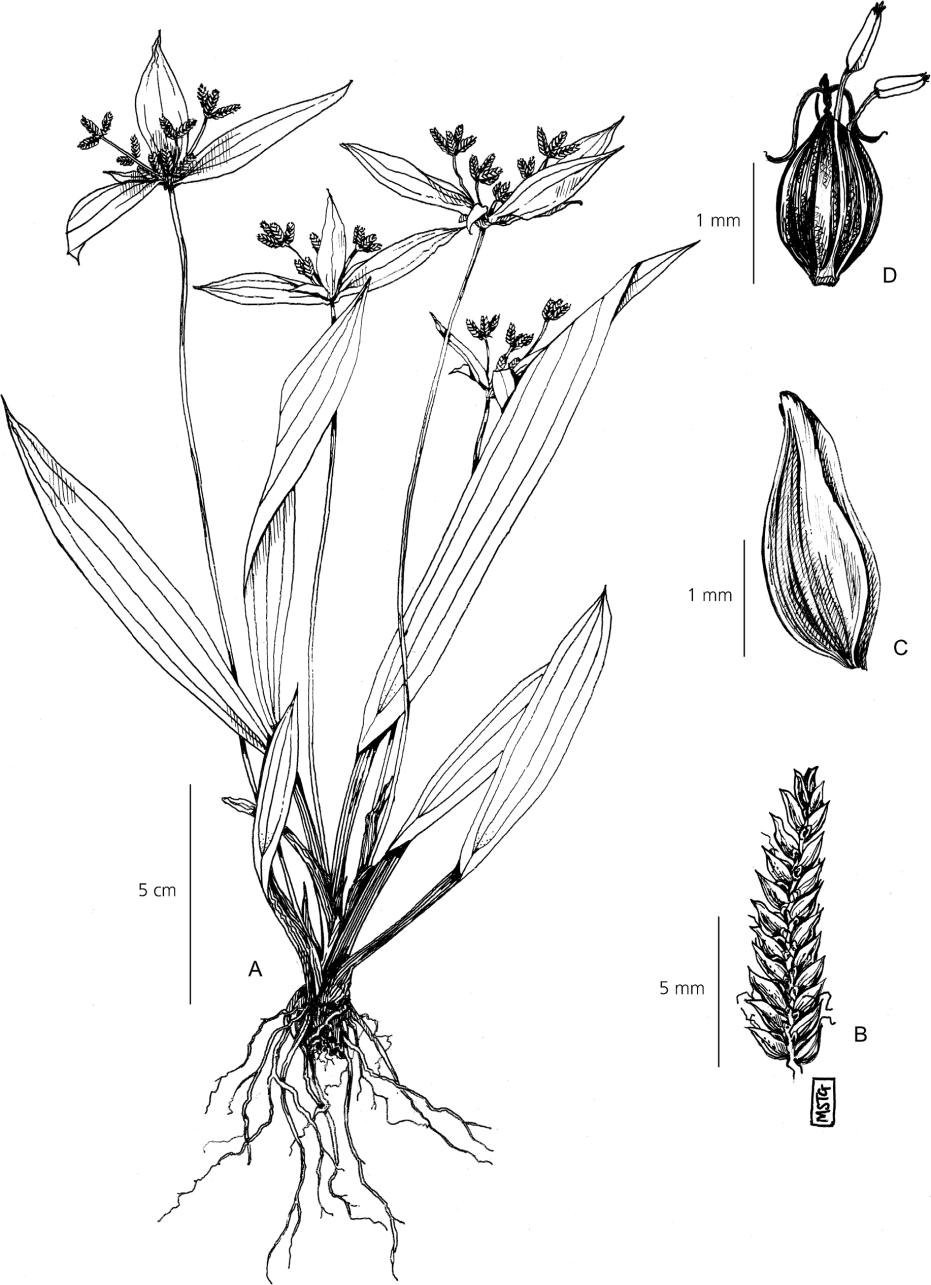
*Cyperus sciaphilus*. **A** habit; **B** spikelet; **C** glume; **D** nutlet. **A** from *Cours* 4842; **B, C, D** from *Beentje* 4829. drawn by m. griffiths.

Herbaceous perennial. *Rhizomes* short, producing slender, red stolons. *Culms* slender, 20 – 30 cm × 1 mm, triangular and ridged. *Leaves* ovate to lanceolate, shorter than the culms, 7 – 15 × 1.5 – 2 cm, smooth, 3-nerved venation runs the length of the leaf blade, leaf margins abruptly fold towards the base, drawing the venation into a sheathed pseudopetiole, 2 – 7 cm × 1 – 3 mm. *Involucral bracts* leaf-like, 5 – 7, spreading away from the inflorescence, variable length up to 10 × 2 cm. *Inflorescence* compound, 2 – 3 × 1.5 – 4 cm anthelate-digitate, 4 – 6 digitate clusters per inflorescence, 2 – 4 spikelets per digitate cluster, each cluster held atop a ray 1 – 5 cm long. *Spikelet*s central-linear or oblong, 4 – 10 × 2 – 3 mm, bearing 6 – 24 flowers. *Glumes* loosely imbricate, spreading, elongated oval and briefly mucronate, 1.5 – 2 × 1 mm, pale green to straw, with many vertical striations. *Stamens* 3, anther tip linear, with minute bristles at the apex. Stigma 3-branched, short, 0.2 mm long, and curled back. *Nutlet* obtuse, 0.8 – 1.5 × 0.7 – 1 mm, trigonous and dark brown, with ellipsoid scales. Fig. 14.

**distribution**. *Cyperus sciaphilus* is endemic to the Central-Eastern escarpment, and Eastern coast of Madagascar. The species is distributed across the entire north-south axis of the island, from the northern Sava region in Antsiranana, through Fianarantsoa, and Toamasina, to the southerly province of Toliara.

**specimens examined.** **madagascar**: Toamasina, Mananara Biosphere Reserve, 9 km W of Antanambe, 170 m, [16°29'S, 49°41'E], 28 Oct. 1994, *Beentje* 4829; Analamazoatra, 1000 m, *Perrier de la Bathie* 15973 (K).

**habitat**. The species typically occurs in the mossy, damp understorey of Madagascan rainforests, as well as freshwater wetlands, rocky slopes and shrublands.

**conservation status**. *Cyperus sciaphilus* has a minimum AOO of 52 km^2^, and an estimated EOO of 157,317 km^2^, above the threshold geographic range for classifying a species as Threatened. However, this is not to say the species is without threat. Like many Madagascan forest endemics, this species is at risk of habitat destruction through agricultural expansion, forest exploitation, and competition with invasive species. All of these threats are forecast to intensify with extended drought periods, brought about by climate change, and the economic devastation left in the wake of COVID-19. However, given its wide geographic range, we assess *C. sciaphilus* as Least Concern LC for the time being.
